# Cell-Associated HIV-1 Unspliced-to-Multiply-Spliced RNA Ratio at 12 Weeks of ART Predicts Immune Reconstitution on Therapy

**DOI:** 10.1128/mBio.00099-21

**Published:** 2021-03-09

**Authors:** Mirte Scherpenisse, Neeltje A. Kootstra, Margreet Bakker, Ben Berkhout, Alexander O. Pasternak

**Affiliations:** a Amsterdam UMC, University of Amsterdam, Laboratory of Experimental Virology, Department of Medical Microbiology, Amsterdam, The Netherlands; b Amsterdam UMC, University of Amsterdam, Laboratory of Viral Immune Pathogenesis, Department of Experimental Immunology, Amsterdam, The Netherlands; University of Pittsburgh School of Medicine

**Keywords:** AIDS, cell-associated RNA, human immunodeficiency virus, immune reconstitution, viral persistence, viral reservoirs

## Abstract

Incomplete restoration of CD4^+^ T-cell counts on antiretroviral therapy (ART) is a major predictor of HIV-related morbidity and mortality. To understand the possible mechanisms behind this poor immunological response despite viral suppression, we longitudinally measured more than 50 virological and immunological biomarkers in a cohort of HIV-infected individuals at several time points during the first 96 weeks of virologically suppressive ART. No baseline virological or immunological marker was predictive of the degree of immune reconstitution. However, the cell-associated HIV-1 unspliced-to-multiply-spliced (US/MS) RNA ratio at 12 weeks of ART positively correlated with markers of CD4^+^ T-cell activation and apoptosis and negatively predicted both the absolute and relative CD4^+^ T-cell counts at 48 and 96 weeks. A higher US/MS RNA ratio may reflect the higher frequency of productively infected cells that could exert pressure on the immune system, contributing to persistent immune activation and apoptosis and subsequently to a poor immunological response to ART.

## Abstract (precis)

Human immunodeficiency virus (HIV) infection is currently managed by antiretroviral drugs, which block virus replication and promote immune restoration. However, the latter effect is not universal, with a proportion of infected individuals failing to sufficiently reconstitute their immune function despite a successful virological response to antiretroviral therapy (ART).

## INTRODUCTION

Antiretroviral therapy (ART) leads to fast and durable suppression of the HIV-1 load in plasma and improvement of immune function (1). However, while the former effect is nearly universal in adherent individuals, the latter effect is more variable. Immunological failure on ART is a frequent clinical phenomenon, as different studies estimate that up to 36% of individuals are unable to restore normal CD4^+^ counts despite a successful (as measured by commercial assays) virological response to therapy (2–6). Suboptimal CD4^+^ T-cell restoration is associated with increased AIDS- and non-AIDS-related morbidity and mortality (7–10). Notwithstanding such alarming proportions, there is still no efficient therapeutic strategy to facilitate immune reconstitution.

Both insufficient CD4^+^ T-cell production and excessive CD4^+^ T-cell destruction contribute to suboptimal CD4^+^ T-cell recovery (11–13). Although low levels of naive CD4^+^ T cells have been repeatedly observed in immunological nonresponders (14–20), it is controversial whether insufficient thymic production leads to immunological failure (14, 16, 21–23). Excessive CD4^+^ T-cell destruction is inherently linked to immune activation, as most CD4^+^ T cells die by apoptosis once activated. Individuals on ART have persistently elevated levels of inflammation and T-cell activation (15, 24), and many groups demonstrated increased T-cell activation and apoptosis in immunological failure (15, 16, 19, 22, 25–31). The etiology of persistent immune activation in the context of successful ART is multifactorial and includes microbial translocation and coinfections, among other factors (32, 33). Some groups indeed demonstrated increased levels of microbial translocation markers and more frequent coinfections with hepatitis C virus (HCV) and hepatitis B virus (HBV) in immunological failure (19, 34–37). In addition, HIV persistence appears to be a major factor behind the elevated immune activation and inflammation in ART-treated individuals (38). Residual virus production, observed in most individuals on suppressive ART, may induce immune activation due to the anti-HIV immune response. In poor immunological responders to ART, a correlation was reported between HIV-1 residual viremia and persistent T-cell activation (39). Higher levels of HIV-1 RNA in plasma and cell-associated (CA) HIV-1 RNA and DNA in peripheral blood and gut tissue on ART were shown to correlate with cell-associated and soluble markers of immune activation and/or with reduced CD4^+^ T-cell gains (17, 30, 40–44), although other investigators challenged these findings (16, 21, 45, 46). We previously observed a strong inverse correlation between on-ART levels of CA HIV-1 unspliced (US) RNA and the baseline and on-ART CD4^+^ counts (47). Moreover, CD8^+^ T-cell activation was shown to be strongly increased in immunological failure, suggesting continuous antigenic stimulation (15, 19, 22, 35). In line with this, intensification of suppressive ART with raltegravir reduced immune activation in individuals on long-term ART, with a particular effect on CD8^+^ T-cell activation (48–53), although not all studies observed this effect (54, 55). At the same time, immune activation may also promote HIV-1 persistence by generating new target cells for residual infection, stimulating virus production from latently infected cells, and/or increasing the proliferation of infected cells. Whether immune activation is a cause or a consequence of HIV-1 persistence, or both, is not known. In fact, viral persistence and residual immune activation may fuel each other in a “vicious circle” (33, 38), and both these components can contribute to immunological failure (56).

Notwithstanding the complexity of the biological pathways leading to poor immune recovery, many groups attempted to identify simple correlates and predictors of immune reconstitution on ART. In addition to the studies discussed above, elevated levels of immune proliferation, senescence, and exhaustion as well as percentages of regulatory T cells (T_reg_s) were shown to correlate with poor immune reconstitution (19, 26, 28, 35, 57–59). However, surprisingly few reliable and specific predictive markers of immunological failure have been identified. Many studies demonstrated that older age is predictive of a poor immunological response, likely due to lower thymic activity (2–4, 14, 34, 37, 60–64). Some but not all studies also found male gender to be associated with a lower chance of achieving CD4^+^ T-cell recovery (61, 62, 65, 66). It is firmly established that a higher pre-ART CD4^+^ count is a major determinant of higher CD4^+^ counts later on ART, but it is unclear whether it also predicts higher relative CD4^+^ T-cell gains (4, 5, 34). In fact, some studies observed an association of higher pre-ART CD4^+^ counts with lower relative CD4^+^ T-cell gains (14, 61). A higher pre-ART CD4/CD8 cell ratio is predictive of better immune reconstitution on ART (37, 67). A lower pre-ART plasma viral load (VL) has been shown by many groups to predict lower relative CD4^+^ T-cell gains (2, 4, 34, 37, 61–63, 65, 66, 68, 69). The biology behind this paradoxical effect is unclear, but one explanation could be the increased redistribution of memory CD4^+^ T cells from lymphoid tissues to the periphery after ART initiation in individuals with higher pre-ART viral loads. Apart from these markers, higher pre-ART CD8^+^ T-cell activation has been shown by two groups to predict lower CD4^+^ T-cell recovery (70, 71), but another group did not observe that effect (72). Pre-ART naive CD4^+^ T-cell percentages, naive/effector memory CD4^+^ T-cell ratios, and activity of the immunoregulatory kynurenine pathway of tryptophan catabolism have also been shown to predict CD4^+^ count normalization (73–75).

Although the clinical management of HIV-1 infection needs validated biomarkers that can predict the degree of immune reconstitution on ART, such biomarkers are currently scarce. Here, we longitudinally measured the levels of more than 50 virological and immunological biomarkers in a cohort of HIV-infected individuals at several time points during the first 96 weeks of virologically suppressive ART and assessed the predictive values of biomarkers measured at baseline (pre-ART) and early on ART for the immunological response to therapy. Although no baseline virological or immunological marker predicted the degree of immune reconstitution, the CA HIV-1 unspliced-to-multiply-spliced (US/MS) RNA ratio at 12 weeks of ART was the only marker that negatively predicted both the absolute and relative CD4^+^ T-cell counts at both 48 and 96 weeks of ART. Moreover, the same marker positively correlated with markers of CD4^+^ T-cell activation and apoptosis at 12 weeks of ART. Our results underscore the influence of residual HIV-1 activity on immunological recovery on virologically suppressive ART.

## RESULTS

### Study participants and measurements.

**FIG 1:**
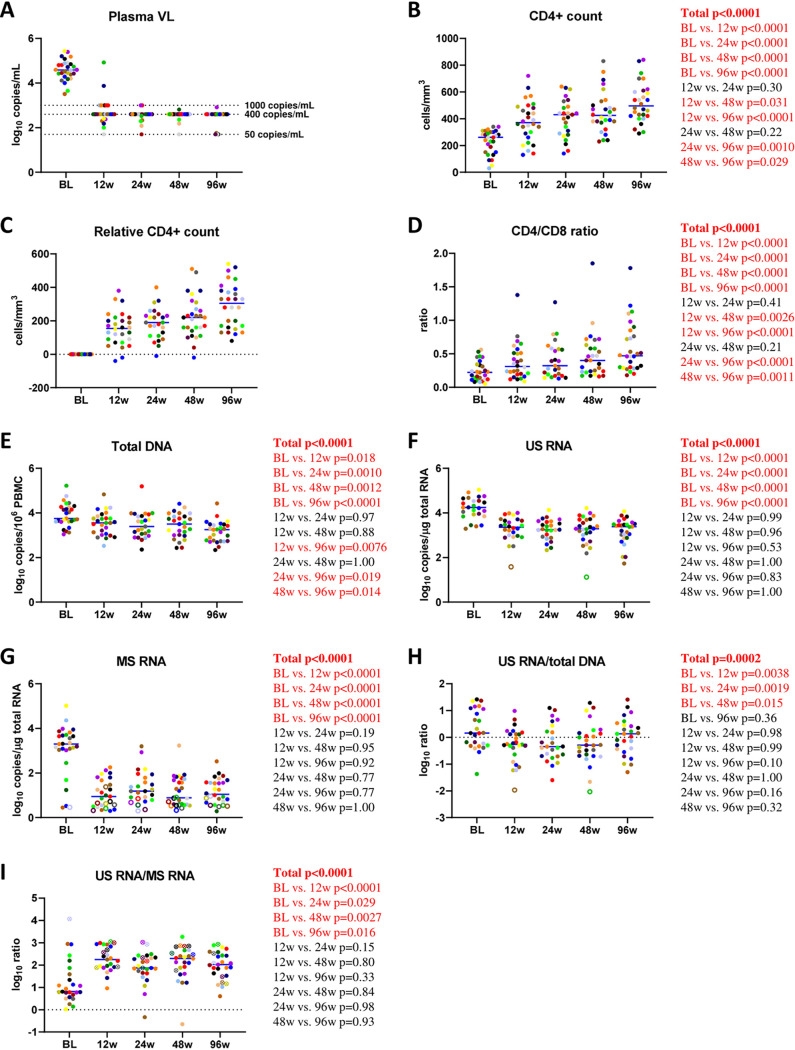
Longitudinal dynamics of CD4^+^ counts, CD4/CD8 ratios, and virological biomarkers during the first 96 weeks of ART. Participants are color-coded. Open circles depict undetectable values, censored to the detection limits. For the US RNA/total DNA ratios, open circles correspond to the samples where US RNA was undetectable and censored to the detection limits; because total DNA in these samples was detectable, these circles depict the upper limits of the US RNA/total DNA ratios. For the US/MS RNA ratios, open crossed circles correspond to the samples where MS RNA was undetectable and censored to the detection limits; because US RNA in these samples was detectable, these circles depict the lower limits of the US/MS RNA ratios. Two samples where both US and MS RNA were undetectable were excluded from the US/MS ratio calculations. For plasma viral load (VL), limits of detection of the commercial assays are shown with dashed lines. Repeated-measures mixed-effects *P* values (representing the significance values of the change of the parameter between the time points) as well as *P* values of pairwise comparisons between the biomarker values at different time points (adjusted to account for multiple comparisons) are depicted to the right of the corresponding graphs. Significant effects are shown in red. BL, baseline.

Total HIV-1 DNA and CA HIV-1 US and MS RNAs; markers of CD4^+^ and CD8^+^ T-cell activation, proliferation, senescence, apoptosis, exhaustion, and thymic migration; and CD4^+^ and CD8^+^ T-cell subsets were longitudinally measured in archival peripheral blood mononuclear cell (PBMC) samples obtained from a retrospective cohort of 28 HIV-infected individuals. Biomarkers were measured at 0, 12, 24, 48, and 96 weeks of virologically suppressive ART. Baseline and treatment characteristics of the study participants are shown in Table S1 in the supplemental material. By week 24 of ART, all participants had achieved suppressed plasma viral loads and maintained viral suppression throughout week 96 of ART, with the exception of occasional “blips” in four participants that remained at <1,000 copies/ml (Fig. 1A; Fig. S1A). CD4^+^ counts increased from a median of 260 (interquartile range [IQR], 150 to 300) cells/mm^3^ at baseline to 425 (378 to 508) cells/mm^3^ at 48 weeks and 495 (420 to 603) cells/mm^3^ at 96 weeks of ART (Fig. 1B; Fig. S1B). The relative gain in CD4^+^ counts at week 48 ranged from −20 to 510 cells/mm^3^ with a median of 220 cells/mm^3^, and those at week 96 ranged from 80 to 540 cells/mm^3^ with a median of 305 cells/mm^3^ (Fig. 1C). The CD4/CD8 cell ratio increased from 0.22 (0.12 to 0.33) at baseline to 0.40 (0.23 to 0.59) at 48 weeks and 0.47 (0.30 to 0.78) at 96 weeks of ART (Fig. 1D).

10.1128/mBio.00099-21.1 FIG S1 Longitudinal changes of plasma viral load (VL) and CD4^+^ counts during the first 96 weeks of ART. Lines correspond to individual participants and are color-coded. For plasma VL, limits of detection of the commercial assays are shown with dashed lines. Download FIG S1, PDF file, 0.04 MB. Copyright © 2021 Scherpenisse et al. 2021 Scherpenisse et al. https://creativecommons.org/licenses/by/4.0/ This content is distributed under the terms of the Creative Commons Attribution 4.0 International license.

10.1128/mBio.00099-21.6 TABLE S1 Baseline and treatment characteristics of the study participants (*n* = 28). Download Table S1, PDF file, 0.03 MB. Copyright © 2021 Scherpenisse et al. 2021 Scherpenisse et al. https://creativecommons.org/licenses/by/4.0/ This content is distributed under the terms of the Creative Commons Attribution 4.0 International license.

### Virological biomarkers.

Total HIV-1 DNA levels gradually diminished during the first 96 weeks of ART (median change between baseline and week 96, 0.83 [IQR, 0.41 to 0.96] log_10_ copies/million PBMCs) (Fig. 1E). In contrast, US and MS RNA levels dropped during the first 12 weeks of ART but remained relatively stable afterward (Fig. 1F and G). While US RNA levels dropped by a median of 0.92 (0.75 to 1.13) log_10_ copies/μg total RNA between baseline and 12 weeks of ART, the median drop in MS RNA levels in the same period was 2.13 (1.63 to 2.70) log_10_ copies/μg total RNA. At all time points during ART, a gradient of the relative changes of CA HIV-1 DNA and RNA species from the baseline was observed: the change in total DNA was the smallest, followed by the change in US RNA, while the change in MS RNA was the most prominent (Fig. S2). Finally, while the US RNA/total DNA ratio initially diminished during the first 24 weeks of ART but subsequently increased to pre-ART levels by week 96, the US/MS RNA ratio sharply increased during the first 12 weeks of ART and remained stable afterward (Fig. 1H and I).

10.1128/mBio.00099-21.2 FIG S2 Comparisons of the relative changes of virological biomarkers from the baseline at 12, 24, 48, and 96 weeks of ART. Participants are color-coded. Repeated-measures mixed-effects *P* values as well as *P* values of pairwise comparisons between the biomarkers are depicted to the right of the corresponding graphs. Significant effects are shown in red. Download FIG S2, PDF file, 0.06 MB. Copyright © 2021 Scherpenisse et al. 2021 Scherpenisse et al. https://creativecommons.org/licenses/by/4.0/ This content is distributed under the terms of the Creative Commons Attribution 4.0 International license.

### T-cell subsets.

**FIG 2:**
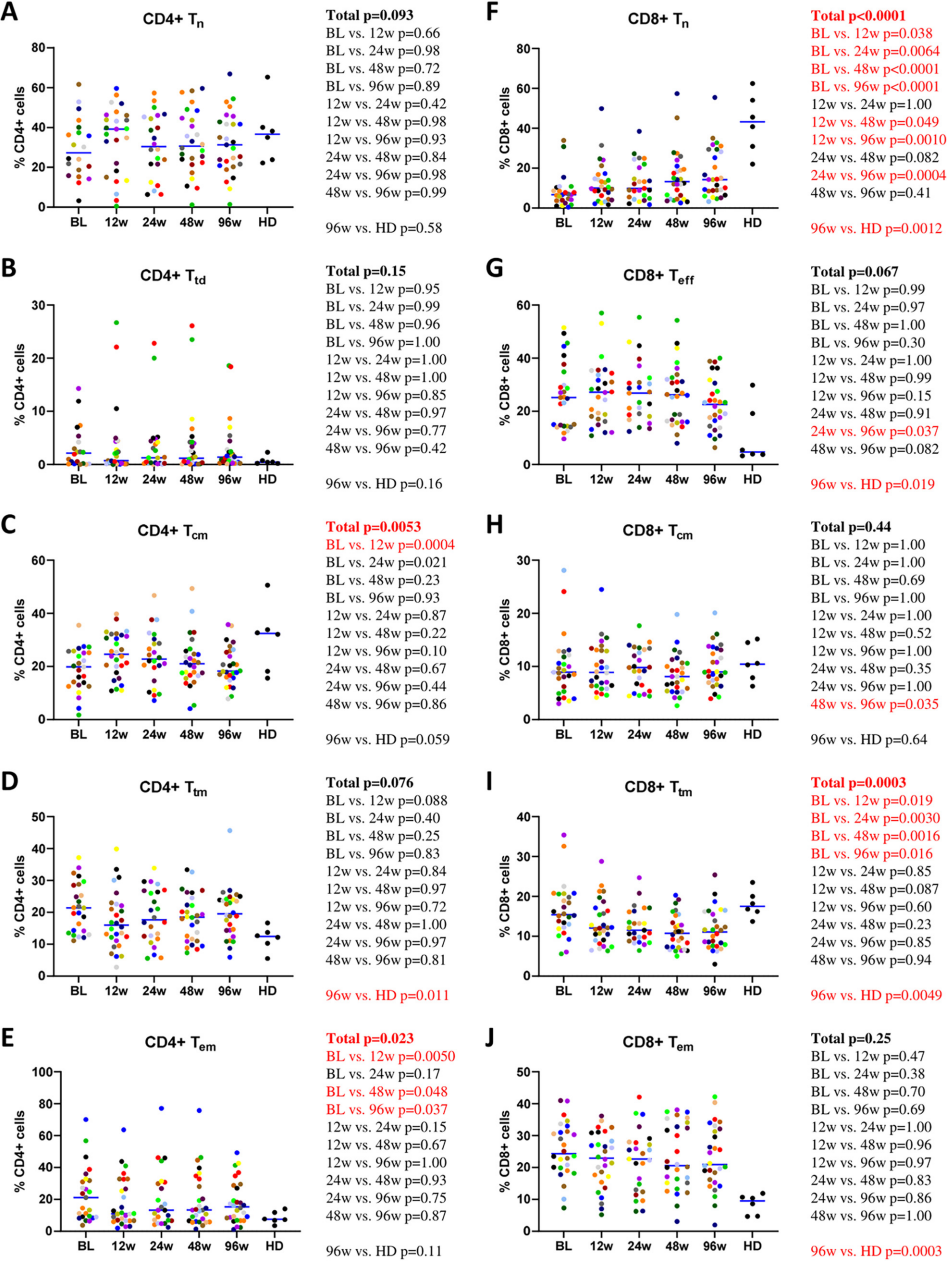
Longitudinal dynamics of CD4^+^ and CD8^+^ T-cell subsets during the first 96 weeks of ART. Participants are color-coded. Repeated-measures mixed-effects *P* values (representing the significance values of the change of the parameter between the time points) as well as *P* values of pairwise comparisons between the biomarker values at different time points (adjusted to account for multiple comparisons) and of comparisons between 96-week values of HIV-infected participants and those of healthy donors (HD) are depicted to the right of the corresponding graphs. Significant effects are shown in red.

CD4^+^ and CD8^+^ T cells were analyzed by flow cytometry as described previously by Chomont et al. (76) to obtain the following subsets: naive (T_n_) (CD45RA^+^ CD27^+^ CCR7^+^), central memory (T_cm_) (CD45RA^−^ CD27^+^ CCR7^+^), transitional memory (T_tm_) (CD45RA^−^ CD27^+^ CCR7^−^), effector memory (T_em_) (CD45RA^−^ CD27^−^ CCR7^−^), as well as terminally differentiated CD4^+^ T cells and effector CD8^+^ T cells (T_td_ and T_eff_, respectively) (CD45RA^+^ CD27^−^ CCR7^−^). The last subset of CD4^+^ and CD8^+^ T cells is also known as effector memory cells reexpressing CD45RA (T_emra_). We determined the percentages of each subset within the total CD4^+^ or CD8^+^ T cells. Different dynamics of CD4^+^ and CD8^+^ T-cell subsets on ART were observed. Changes in the frequencies of CD4^+^ T-cell subsets on ART were minimal, with the exception of T_em_ cells, which diminished from baseline onward (Fig. 2A to E). Frequencies of the CD4^+^ memory T-cell subsets did not reach the levels of healthy donors (HDs) during 96 weeks of ART. In contrast to CD4^+^ T_n_ cells that were stable, CD8^+^ T_n_ cells increased on ART but still did not reach the level of HDs. CD8^+^ T_eff_ and CD8^+^ T_em_ cells were stable and did not normalize on ART. CD8^+^ T_cm_ cells were also stable, but no difference was observed compared to HDs, while CD8^+^ T_tm_ cells diminished on ART to below the level of HDs (Fig. 2F to J).

We also assessed a number of additional CD4^+^ and CD8^+^ T-cell subsets. First, we measured the percentages of CD4^+^ and CD8^+^ T cells expressing Ki67, a marker of T-cell proliferation. While CD4^+^/Ki67^+^ cells remained stable, CD8^+^/Ki67^+^ cells diminished on ART, although neither subset reached the levels of HDs (Fig. S3A and B). Second, recent thymic emigrants (RTEs) were defined based on the expression of CD31 (77–79) as either CD4^+^/CD45RA^+^/CD31^+^ cells or a CD31-positive subset of CD4^+^ T_n_ cells. RTEs were stable during ART (Fig. S3C and D). Third, we measured the percentages of regulatory T cells (T_reg_s), defined as CD4^+^/CD25^+^/FoxP3^+^ cells. T_reg_s diminished on ART, especially during the first 12 weeks, but did not completely normalize (Fig. S3E).

10.1128/mBio.00099-21.3 FIG S3 Longitudinal dynamics of markers of CD4^+^ and CD8^+^ T-cell proliferation, recent thymic emigrants, and regulatory T cells during the first 96 weeks of ART. Participants are color-coded. Repeated-measures mixed-effects *P* values as well as *P* values of pairwise comparisons between the biomarker values at different time points and of comparisons between 96-week values of HIV-infected participants and those of healthy donors (HD) are depicted to the right of the corresponding graphs. Significant effects are shown in red. Download FIG S3, PDF file, 0.09 MB. Copyright © 2021 Scherpenisse et al. 2021 Scherpenisse et al. https://creativecommons.org/licenses/by/4.0/ This content is distributed under the terms of the Creative Commons Attribution 4.0 International license.

### T-cell activation, senescence, and exhaustion.

**FIG 3:**
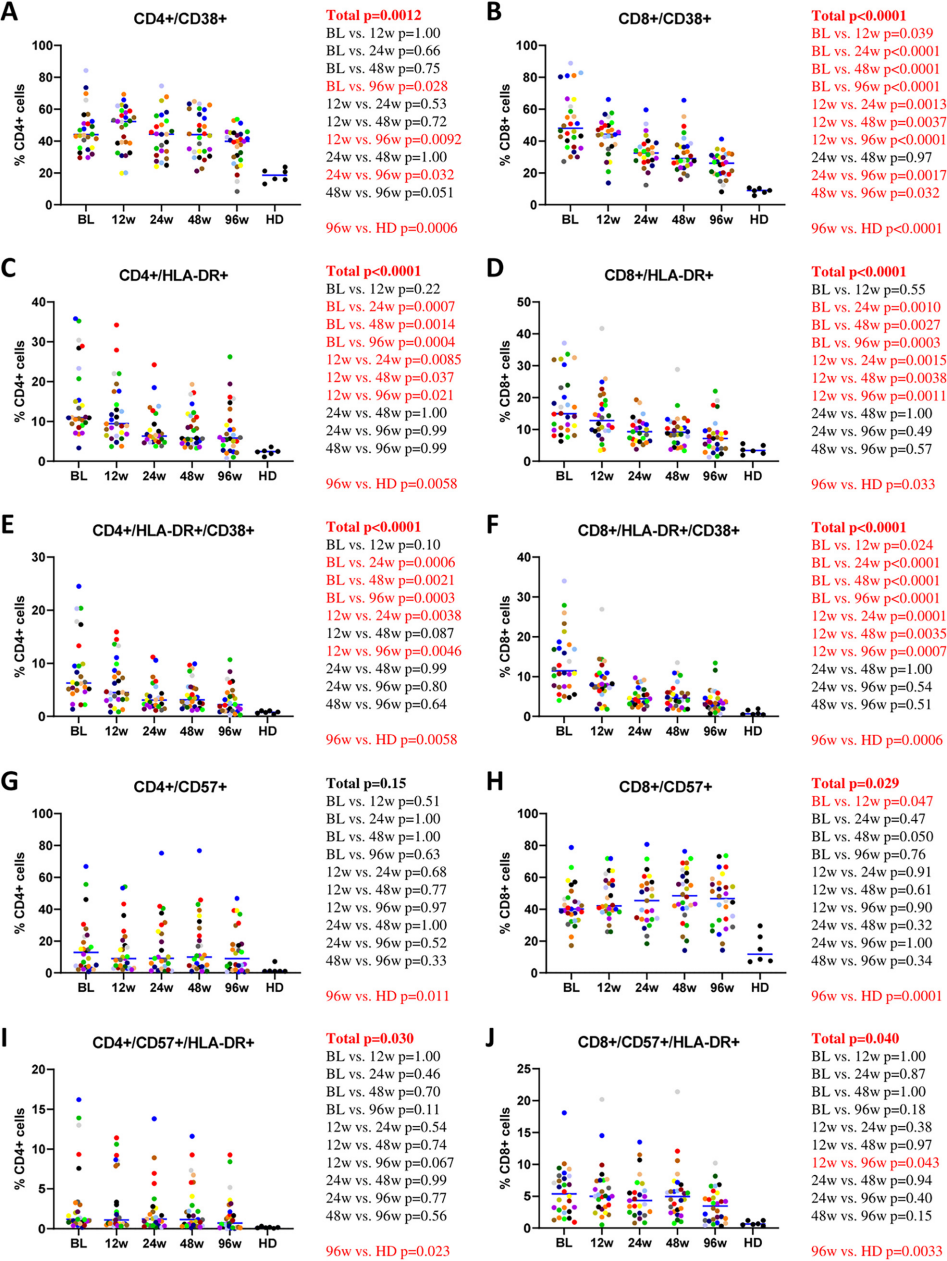
Longitudinal dynamics of markers of CD4^+^ and CD8^+^ activation and senescence during the first 96 weeks of ART. Participants are color-coded. Repeated-measures mixed-effects *P* values (representing the significance values of the change of the parameter between the time points) as well as *P* values of pairwise comparisons between the biomarker values at different time points (adjusted to account for multiple comparisons) and of comparisons between 96-week values of HIV-infected participants and those of healthy donors (HD) are depicted to the right of the corresponding graphs. Significant effects are shown in red.

CD4^+^ and CD8^+^ T-cell activation was determined by measuring the percentages of CD38^+^, HLA-DR^+^, and CD38^+^/HLA-DR^+^ T cells. Levels of both CD4^+^ and CD8^+^ activation steadily diminished but did not completely normalize during the first 96 weeks of ART (Fig. 3A to F). In contrast, levels of immune senescence, defined as percentages of CD4^+^ and CD8^+^ cells expressing CD57 (80), were stable and also did not reach the levels of HDs (Fig. 3G and H). Levels of T cells coexpressing CD57 and HLA-DR were relatively stable as well and did not normalize on ART (Fig. 3I and J). We also determined the levels of immune exhaustion by measuring the percentages of CD4^+^ and CD8^+^ cells expressing CTLA-4 and PD-1 as well as cells coexpressing these molecules (81, 82). All these T-cell subsets remained stable on ART, with the exception of CD8^+^/PD-1^+^ cells, which slightly diminished, and did not achieve the levels of HDs (Fig. S4A to F). The same dynamics was observed for cells coexpressing markers of immune senescence and exhaustion (CD57^+^/PD-1^+^) (Fig. S4G and H).

10.1128/mBio.00099-21.4 FIG S4 Longitudinal dynamics of markers of CD4^+^ and CD8^+^ exhaustion during the first 96 weeks of ART. Participants are color-coded. Repeated-measures mixed-effects *P* values as well as *P* values of pairwise comparisons between the biomarker values at different time points and of comparisons between 96-week values of HIV-infected participants and those of healthy donors (HD) are depicted to the right of the corresponding graphs. Significant effects are shown in red. Download FIG S4, PDF file, 0.1 MB. Copyright © 2021 Scherpenisse et al. 2021 Scherpenisse et al. https://creativecommons.org/licenses/by/4.0/ This content is distributed under the terms of the Creative Commons Attribution 4.0 International license.

### T-cell apoptosis.

**FIG 4:**
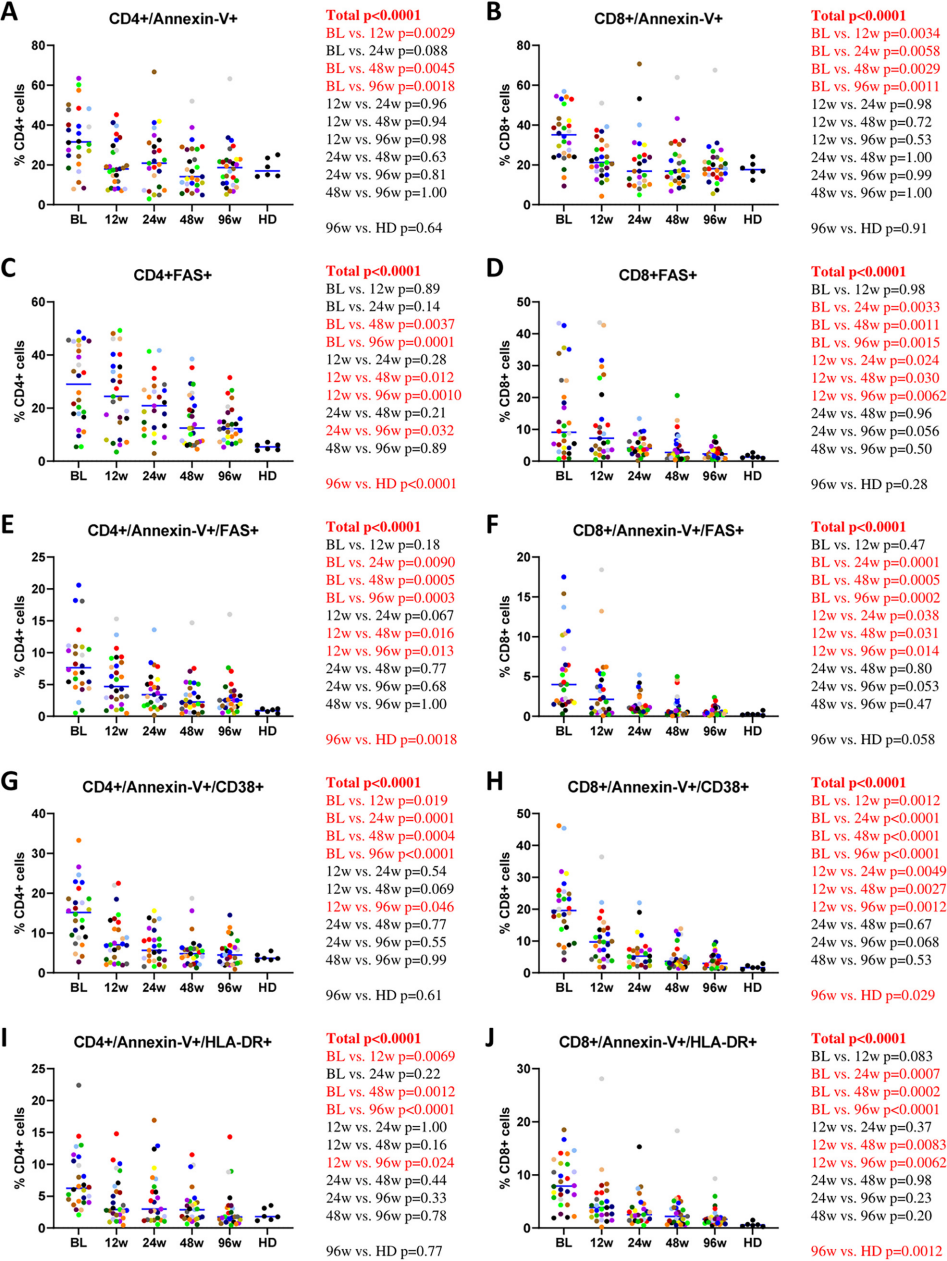
Longitudinal dynamics of markers of CD4^+^ and CD8^+^ apoptosis during the first 96 weeks of ART. Participants are color-coded. Repeated-measures mixed-effects *P* values (representing the significance values of the change of the parameter between the time points) as well as *P* values of pairwise comparisons between the biomarker values at different time points (adjusted to account for multiple comparisons) and of comparisons between 96-week values of HIV-infected participants and those of healthy donors (HD) are depicted to the right of the corresponding graphs. Significant effects are shown in red.

Levels of CD4^+^ and CD8^+^ T-cell apoptosis were determined by either single or double staining with annexin V and/or Fas antibody (16, 27). Similar to the levels of immune activation, the percentages of apoptotic cells steadily diminished on ART, but although the levels of annexin V-positive (annexin V^+^) CD4^+^ and CD8^+^ cells normalized on therapy, Fas^+^ and annexin V^+^/Fas^+^ cells did not achieve the levels of HDs (Fig. 4A to F). We also determined the percentages of apoptotic cells expressing markers of immune activation (CD38 or HLA-DR). Both annexin V^+^/CD38^+^ and annexin V^+^/HLA-DR^+^ CD4^+^ and CD8^+^ cells demonstrated a steady decrease on ART, but while the levels of apoptotic CD4^+^ cells expressing markers of activation normalized by week 96, the corresponding CD8^+^ cells still did not achieve the levels of HDs (Fig. 4G to J).

### Correlations between virological and immunological markers.

**FIG 5:**
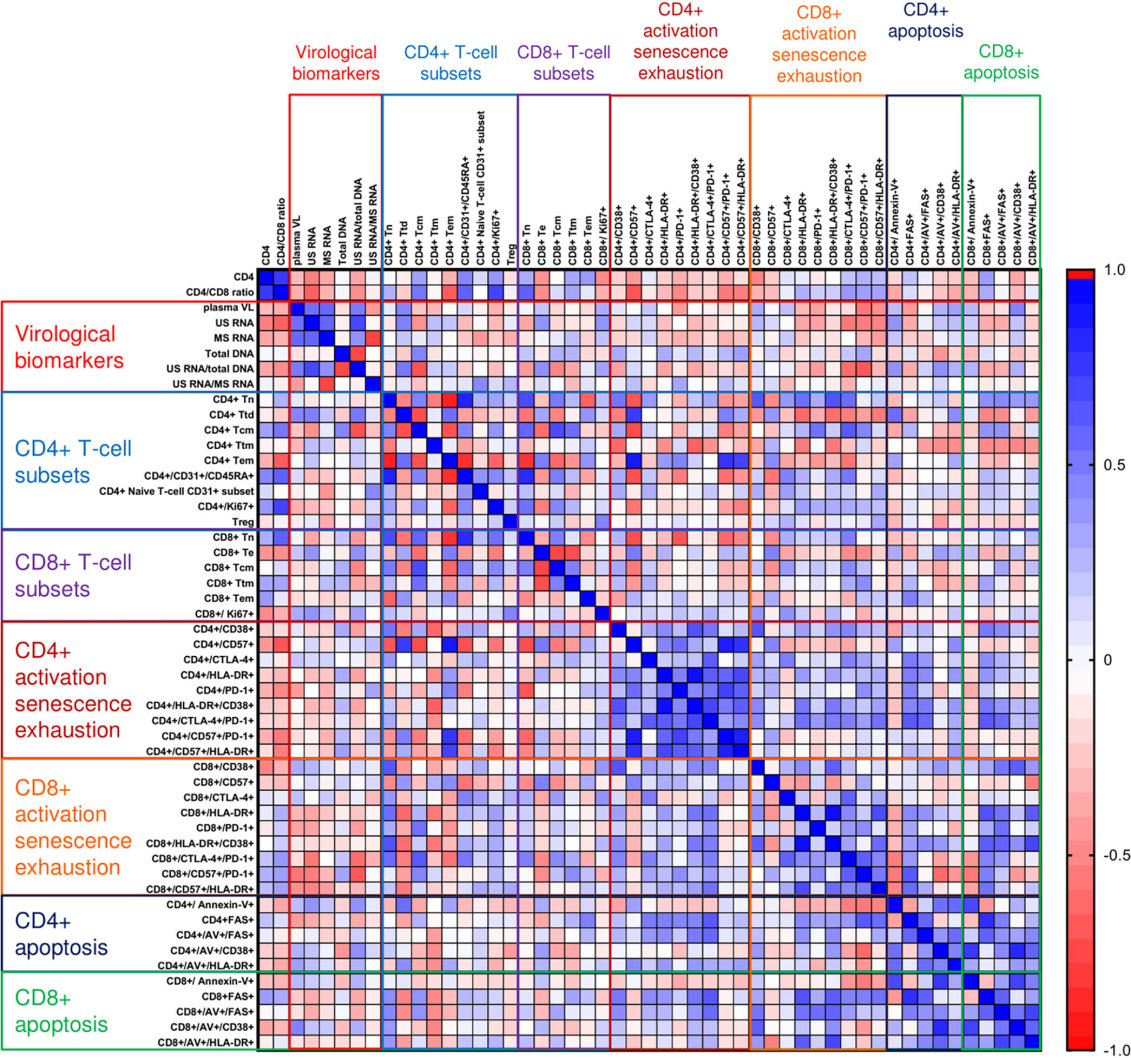
Spearman correlogram of CD4^+^ counts, CD4/CD8 ratios, and virological and immunological biomarkers at baseline. A heat map is used to indicate the strengths of associations between biomarkers. Red indicates a negative correlation, and blue indicates a positive correlation.

**FIG 6:**
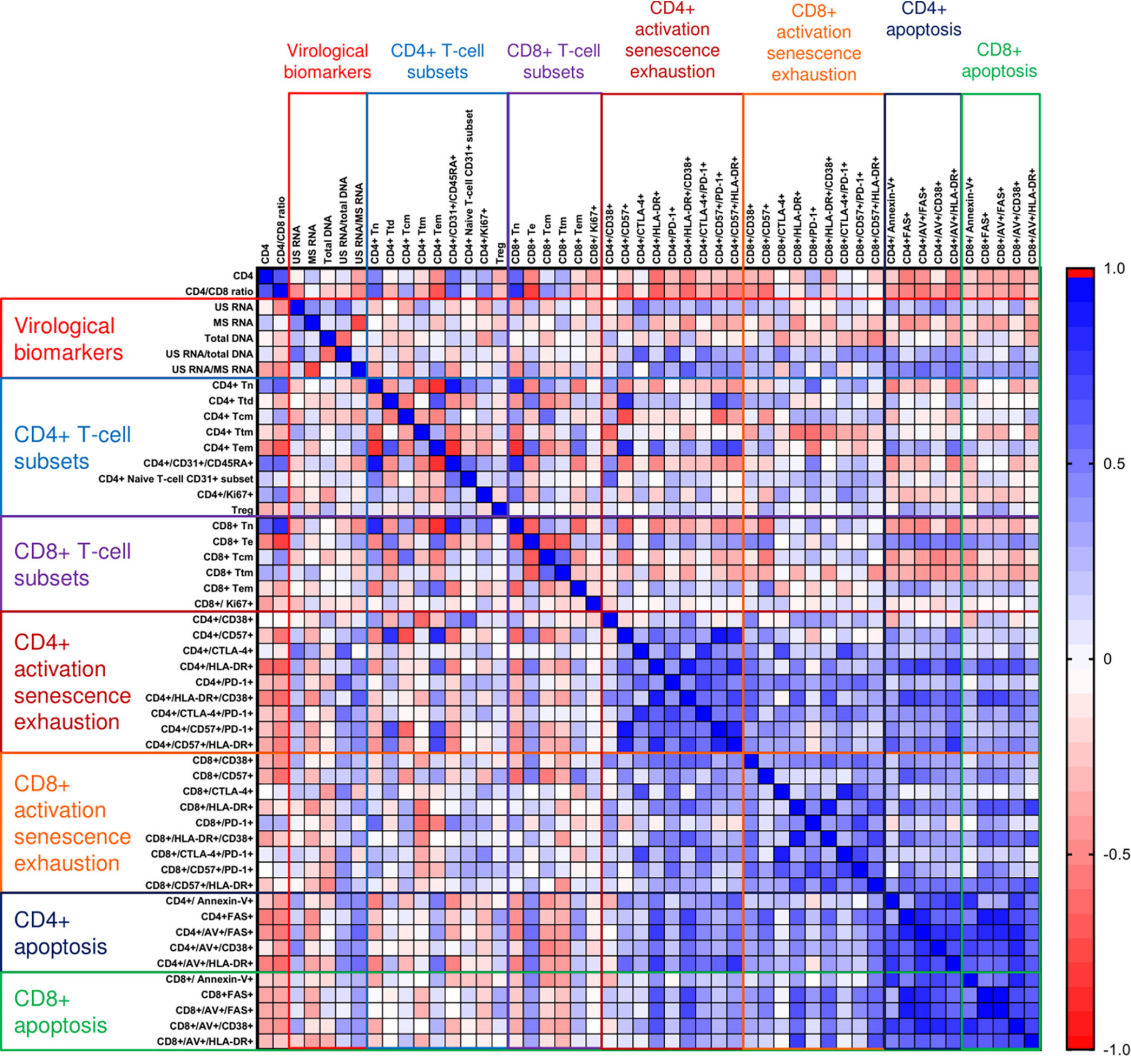
Spearman correlogram of CD4^+^ counts, CD4/CD8 ratios, and virological and immunological biomarkers at 12 weeks of ART. A heat map is used to indicate the strengths of associations between biomarkers. Red indicates a negative correlation, and blue indicates a positive correlation.

At baseline and at each time point during ART, we determined pairwise correlations between CD4^+^ counts, CD4/CD8 ratios, and all virological and immunological biomarkers. Figures 5 and 6 and Fig. S5A to C show Spearman correlograms per time point (for the rho and *P* values, see the supplemental data set available at https://doi.org/10.6084/m9.figshare.12942719). In general, different markers of activation and apoptosis strongly positively correlated with each other at all time points, for both CD4^+^ and CD8^+^ cells, but correlations between virological and immunological markers were very different in untreated infection and early on ART. At baseline, no strong positive correlations were observed between virological markers and markers of immune activation or apoptosis, but some markers of CD8^+^ cell exhaustion such as CD8^+^/CD57^+^/PD-1^+^ cell percentages negatively correlated with plasma viral load, US RNA, and the US RNA/total DNA ratio. In addition, T_cm_ percentages negatively correlated with the US RNA/total DNA ratio (Fig. 5). However, at week 12, many markers of CD4^+^ and CD8^+^ cell activation, exhaustion, and apoptosis strongly positively correlated with virological markers, in particular with the US RNA/total DNA and US/MS RNA ratios. The strongest correlations were observed between the US RNA/total DNA ratios and the percentages of CD4^+^ cells expressing CTLA-4 (rho = 0.64; *P* = 4.5 × 10^−4^) and PD-1 (rho = 0.63; *P* = 5.8 × 10^−4^) and between the US/MS RNA ratios and the percentages of CD4^+^ cells coexpressing CD38 and HLA-DR (rho = 0.63; *P* = 7.8 × 10^−4^) and annexin V^+^/Fas^+^ CD4^+^ cells (rho = 0.59; *P* = 0.0023) (Fig. 6). In general, correlations between virological and immunological markers became weaker at the subsequent time points on therapy, but US RNA strongly positively correlated with CD4^+^/CTLA-4^+^ and CD4^+^/PD-1^+^ cell percentages at 24 and 96 weeks of ART (Fig. S5A to C).

10.1128/mBio.00099-21.5 FIG S5 Spearman correlograms of CD4^+^ counts, CD4/CD8 ratios, and virological and immunological biomarkers at 24 weeks (A), 48 weeks (B), and 96 weeks (C) of ART. A heat map is used to indicate the strengths of associations between biomarkers. Red indicates a negative correlation, and blue indicates a positive correlation. Download FIG S5, PDF file, 0.3 MB. Copyright © 2021 Scherpenisse et al. 2021 Scherpenisse et al. https://creativecommons.org/licenses/by/4.0/ This content is distributed under the terms of the Creative Commons Attribution 4.0 International license.

We also performed correlation analyses between the time points for every biomarker separately (see the supplemental figure available at https://doi.org/10.6084/m9.figshare.12942848). In general, these correlations were overwhelmingly positive, but the strengths of the correlations varied between biomarkers. The CD4/CD8 ratio correlated strongly between time points (minimal rho = 0.83), but correlations of the CD4^+^ count were weaker. Among the virological markers, US RNA demonstrated the strongest correlations between time points. Among the T-cell subsets, we observed strong correlations in CD4^+^ and CD8^+^ T_n_, CD4^+^ T_em_, and CD4^+^ T_td_ cells and RTEs (CD4^+^/CD45RA^+^/CD31^+^), while correlations of CD4^+^ and CD8^+^ Ki67^+^ cells, as well as T_reg_s, were weak. Weak correlations were also observed between markers of CD4^+^ and CD8^+^ T-cell activation and especially exhaustion, where some pairwise correlations, in particular correlations between baseline and on-ART percentages of CD4^+^ and CD8^+^ cells coexpressing CTLA-4 and PD-1, were negative. In contrast, percentages of CD4^+^ and CD8^+^ cells expressing CD57 strongly positively correlated between time points, and so did CD4^+^ cells (but not CD8^+^ cells) coexpressing CD57 and HLA-DR or PD-1. Finally, weaker correlations were observed for markers of T-cell apoptosis and for coexpression of markers of apoptosis and activation.

### Correlations between CD4^+^ counts and other biomarkers.

Table 1 shows correlations between CD4^+^ counts and levels of other biomarkers, per time point. Although a number of biomarkers correlated with CD4^+^ counts at baseline, only the correlation of the CD4/CD8 ratio remained significant after correction for multiple comparisons. However, more biomarkers significantly correlated with CD4^+^ counts on ART, also after correction for multiple comparisons. Apart from the CD4/CD8 ratio, percentages of CD4^+^ T_n_ cells, CD8^+^ T_n_ cells, and RTEs positively correlated with CD4^+^ counts at all or most time points on ART, while percentages of CD4^+^ T_em_ cells and markers of CD4^+^ T-cell activation (CD4^+^/HLA-DR^+^ and CD4^+^/CD38^+^/HLA-DR^+^) and apoptosis (CD4^+^/annexin V^+^/HLA-DR^+^) demonstrated a negative correlation. No virological markers correlated with the CD4^+^ count at any on-ART time point, although a negative correlation with US RNA was observed at baseline. We also assessed the correlations of biomarkers, measured at 48 and 96 weeks of ART, with the relative gains in CD4^+^ counts at these time points. After correction for multiple comparisons, only the absolute CD4^+^ counts remained associated with the relative CD4^+^ counts at both 48 and 96 weeks of ART (Table S2).

**TABLE 1:** Biomarkers associated with CD4^+^ counts per time point on ART

Biomarker	Baseline		12 wks of ART		24 wks of ART		48 wks of ART		96 wks of ART	
	Rho	*P*^*a*^	Rho	*P*	Rho	*P*	Rho	*P*	Rho	*P*
CD4/CD8 ratio	0.77	**2.63E−06**	0.66	**1.24E−04**	0.78	**5.17E−06**	0.57	**0.0010**	0.61	**0.0010**

Virological biomarkers										
Plasma VL	−0.27	0.17								
US RNA	−0.50	**0.012**	−0.06	0.77	0.05	0.81	0.01	0.96	−0.06	0.75
MS RNA	−0.35	0.075	0.23	0.24	−0.03	0.90	0.00	0.99	−0.11	0.58
Total DNA	0.03	0.89	−0.05	0.82	−0.20	0.33	−0.32	0.10	−0.08	0.68
US RNA/total DNA ratio	−0.35	0.090	0.12	0.57	0.21	0.31	0.33	0.094	0.11	0.60
US RNA/MS RNA ratio	0.05	0.83	−0.36	0.078	0.02	0.93	−0.18	0.38	0.09	0.65

CD4^+^ T-cell subsets										
CD4^+^ T_n_	−0.03	0.90	0.47	**0.017**	0.69	**4.02E−04**	0.64	**4.22E−04**	0.40	**0.040**
CD4^+^ T_td_	−0.07	0.76	−0.05	0.80	−0.26	0.23	−0.13	0.52	0.10	0.63
CD4^+^ T_cm_	0.36	0.075	0.10	0.62	0.31	0.14	0.01	0.96	0.11	0.59
CD4^+^ T_tm_	−0.05	0.81	−0.15	0.45	−0.38	0.064	−0.34	0.079	−0.48	**0.010**
CD4^+^ T_em_	−0.30	0.15	−0.45	**0.017**	−0.58	**0.0029**	−0.43	**0.022**	−0.29	0.14
CD4^+^/CD31^+^/CD45RA^+^	0.47	**0.018**	0.55	**0.0023**	0.67	**3.53E−04**	0.63	**3.37E−04**	0.32	0.10
CD4^+^ naive T-cell CD31^+^ subset	0.15	0.48	0.20	0.31	0.04	0.87	0.16	0.42	−0.16	0.43
CD4^+^/Ki67^+^	0.40	0.051	0.32	0.10	0.32	0.12	0.32	0.093	0.16	0.42
T_reg_	−0.12	0.53	−0.32	0.11	−0.47	**0.018**	−0.49	**0.0082**	−0.37	0.054

CD8^+^ T-cell subsets										
CD8^+^ T_n_	0.31	0.13	0.60	**8.38E−04**	0.61	**0.0013**	0.59	**9.57E−04**	0.51	0.0051
CD8^+^ T_eff_	−0.42	**0.030**	−0.48	**0.011**	−0.59	**0.0018**	−0.27	0.16	−0.43	**0.022**
CD8^+^ T_cm_	−0.02	0.92	0.13	0.52	0.46	**0.022**	−0.03	0.90	0.31	0.11
CD8^+^ T_tm_	0.34	0.088	0.32	0.093	0.43	**0.030**	−0.15	0.43	−0.02	0.92
CD8^+^ T_em_	0.39	**0.047**	−0.16	0.43	−0.22	0.29	−0.08	0.67	−0.25	0.21
CD8^+^/Ki67^+^	−0.46	**0.017**	−0.38	**0.048**	−0.10	0.64	−0.02	0.91	−0.10	0.62

CD4^+^ activation and exhaustion										
CD4^+^/CD38^+^	−0.20	0.34	0.01	0.95	0.31	0.13	0.36	0.060	0.25	0.21
CD4^+^/CD57^+^	−0.40	**0.044**	−0.23	0.26	−0.42	**0.035**	−0.23	0.24	−0.10	0.62
CD4^+^/CTLA-4^+^	0.09	0.67	0.07	0.74	−0.22	0.29	0.05	0.81	−0.02	0.93
CD4^+^/HLA-DR^+^	−0.19	0.35	−0.56	**0.0019**	−0.63	**7.30E−04**	−0.47	**0.011**	−0.13	0.53
CD4^+^/PD-1^+^	−0.31	0.12	−0.26	0.19	−0.29	0.16	−0.21	0.29	−0.09	0.66
CD4^+^/HLA-DR^+^/CD38^+^	−0.21	0.30	−0.48	**0.0099**	−0.56	**0.0036**	−0.39	**0.038**	−0.23	0.24
CD4^+^/CTLA-4^+^/PD-1^+^	−0.15	0.47	−0.22	0.25	−0.32	0.11	−0.23	0.24	−0.08	0.67
CD4^+^/CD57^+^/PD-1^+^	−0.30	0.13	−0.24	0.23	−0.41	**0.043**	−0.18	0.37	−0.17	0.39
CD4^+^/CD57^+^/HLA-DR^+^	−0.13	0.53	−0.33	0.090	−0.45	**0.023**	−0.44	**0.019**	−0.13	0.51

CD8^+^ activation and exhaustion										
CD8^+^/CD38^+^	−0.48	**0.010**	−0.28	0.14	−0.36	0.079	−0.29	0.14	0.04	0.84
CD8^+^/CD57^+^	−0.20	0.30	−0.32	0.10	−0.43	**0.032**	−0.10	0.60	−0.26	0.18
CD8^+^/CTLA-4^+^	0.12	0.56	0.00	1.00	−0.12	0.58	−0.01	0.94	0.13	0.51
CD8^+^/HLA-DR^+^	0.08	0.67	−0.24	0.22	−0.03	0.87	−0.31	0.11	−0.14	0.47
CD8^+^/PD-1^+^	−0.10	0.63	0.30	0.11	0.33	0.11	0.08	0.68	0.08	0.69
CD8^+^/HLA-DR^+^/CD38^+^	0.00	0.98	−0.39	**0.043**	−0.33	0.11	−0.40	**0.037**	−0.10	0.61
CD8^+^/CTLA-4^+^/PD-1^+^	0.23	0.23	0.07	0.71	−0.10	0.65	0.00	0.99	0.04	0.83
CD8^+^/CD57^+^/PD-1^+^	0.30	0.12	−0.01	0.98	0.10	0.64	0.05	0.78	−0.07	0.72
CD8^+^/CD57^+^/HLA-DR^+^	0.25	0.20	−0.22	0.28	−0.14	0.50	−0.20	0.31	−0.22	0.26

CD4^+^ apoptosis										
CD4^+^/annexin V^+^	−0.16	0.43	−0.21	0.30	−0.43	**0.034**	−0.10	0.62	0.17	0.39
CD4^+^ Fas^+^	0.24	0.25	−0.51	**0.0064**	−0.19	0.37	−0.40	**0.041**	−0.42	**0.026**
CD4^+^/annexin V^+^/Fas^+^	0.08	0.71	−0.43	**0.027**	−0.39	0.054	−0.35	0.076	−0.17	0.39
CD4^+^/annexin V^+^/CD38^+^	−0.20	0.34	−0.09	0.64	−0.29	0.16	0.25	0.20	−0.08	0.67
CD4^+^/annexin V^+^/HLA-DR^+^	−0.20	0.32	−0.49	**0.0088**	−0.46	**0.021**	−0.46	**0.016**	−0.24	0.22

CD8^+^ apoptosis										
CD8^+^/annexin V^+^	−0.25	0.20	−0.18	0.36	−0.32	0.11	−0.17	0.39	0.31	0.11
CD8^+^ Fas^+^	0.38	**0.048**	−0.35	0.076	0.13	0.54	−0.17	0.40	−0.10	0.62
CD8^+^/annexin V^+^/Fas^+^	−0.03	0.87	−0.34	0.087	0.00	1.00	−0.34	0.085	−0.05	0.79
CD8^+^/annexin V^+^/CD38^+^	−0.34	0.07	−0.37	0.054	−0.45	**0.024**	−0.19	0.34	0.06	0.75
CD8^+^/annexin V^+^/HLA-DR^+^	−0.07	0.73	−0.27	0.17	−0.16	0.44	−0.30	0.12	0.04	0.84

a Significant *P* values are shown in boldface type, and those that remained significant after Benjamini-Hochberg correction for multiple comparisons are underlined.

10.1128/mBio.00099-21.7 TABLE S2 Biomarkers measured at 48 and 96 weeks of ART associated with the relative CD4^+^ counts at the corresponding time points. Download Table S2, PDF file, 0.03 MB. Copyright © 2021 Scherpenisse et al. 2021 Scherpenisse et al. https://creativecommons.org/licenses/by/4.0/ This content is distributed under the terms of the Creative Commons Attribution 4.0 International license.

### Predictive markers of the immunological response on ART.

We next assessed the predictive value of virological and immunological biomarkers, measured at baseline and early on ART, for the absolute CD4^+^ count at 48 and 96 weeks of ART as well as for the relative gain in the CD4^+^ count in the first 48 and 96 weeks of ART. Age, ART composition, and treatment experience prior to the start of combination ART were not associated with any of the endpoints (see the supplemental table available at https://doi.org/10.6084/m9.figshare.12942875 and the supplemental figure available at https://doi.org/10.6084/m9.figshare.12942851). At baseline, several biomarkers were significantly predictive of the absolute CD4^+^ count at 48 weeks of ART (Table S3), but only the baseline CD4^+^ count remained significantly predictive after correction for multiple comparisons (rho = 0.64; *P* = 2.2 × 10^−4^). Some baseline markers were predictive of the absolute CD4^+^ count at 96 weeks and of the relative CD4^+^ count gain by 48 and 96 weeks of ART, but the significance was lost after correction for multiple comparisons (Table S3).

10.1128/mBio.00099-21.8 TABLE S3 Predictive values of baseline biomarkers for the absolute and relative CD4^+^ counts at 48 and 96 weeks of ART. Download Table S3, PDF file, 0.03 MB. Copyright © 2021 Scherpenisse et al. 2021 Scherpenisse et al. https://creativecommons.org/licenses/by/4.0/ This content is distributed under the terms of the Creative Commons Attribution 4.0 International license.

**FIG 7:**
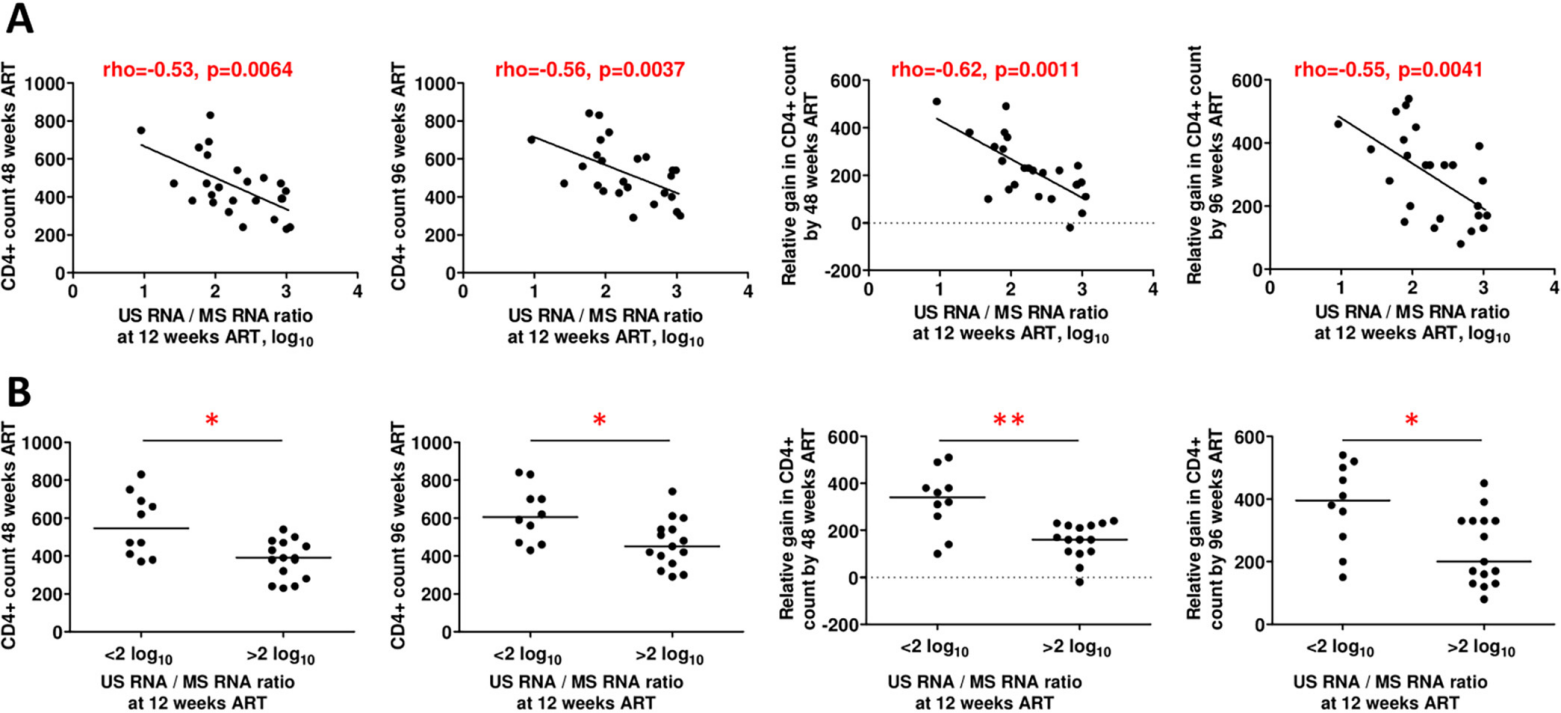
The CA HIV-1 US/MS RNA ratio predicts the immunological response to ART. (A) Spearman correlations between the US/MS RNA ratio at 12 weeks of ART and absolute and relative CD4^+^ counts at 48 and 96 weeks of ART. (B) Comparisons of immunological responses to ART between participants with high and those with low values of US/MS RNA ratios at 12 weeks of ART. *, 0.01 < *P* < 0.05; **, 0.001 < *P* < 0.01.

In contrast, at 12 weeks of ART, a number of markers predicted the absolute and relative CD4^+^ counts at 48 and 96 weeks of ART (Table 2). After correction for multiple comparisons, the absolute CD4^+^ count at 48 weeks was positively predicted by the CD4^+^ count, the CD4/CD8 ratio, CD4^+^ T_n_ cells, CD8^+^ T_n_ cells, and RTEs and negatively predicted by the US/MS RNA ratio. The absolute CD4^+^ count at 96 weeks was positively predicted by the CD4^+^ count and the CD4/CD8 ratio and negatively predicted by the US/MS RNA ratio and CD4^+^/HLA-DR^+^ and CD4^+^/CD38^+^/HLA-DR^+^ cell percentages. A relative gain in the CD4^+^ count by 48 weeks was positively predicted by MS RNA and negatively predicted by the US/MS RNA ratio. A relative gain in the CD4^+^ count by 96 weeks was negatively predicted by the US/MS ratio and CD4^+^/CD38^+^/HLA-DR^+^, CD8^+^/HLA-DR^+^, and CD8^+^/CD38^+^/HLA-DR^+^ cell percentages. Thus, the US/MS RNA ratio was the only marker that remained significantly predictive of all four endpoints after correction for multiple comparisons (Fig. 7A). Participants with 12-week US/MS RNA ratios of <100 achieved significantly higher absolute and relative CD4^+^ counts at weeks 48 and 96 than participants with US/MS RNA ratios of >100 (Fig. 7B). Notably, the 12-week US/MS RNA ratio did not correlate with the baseline CD4^+^ count (rho = −0.15; *P* = 0.46).

**TABLE 2:** Predictive value of biomarkers measured at 12 weeks of ART for the absolute and relative CD4^+^ counts at 48 and 96 weeks of ART

Biomarker	CD4^+^ count at 48 wks		Relative CD4^+^ count at 48 wks		CD4^+^ count at 96 wks		Relative CD4^+^ count at 96 wks	
	Rho	*P*^*a*^	Rho	*P*	Rho	*P*	Rho	*P*
CD4^+^ count	**0.65**	**1.68E−04**	0.16	0.43	**0.59**	**0.0010**	0.12	0.53

CD4/CD8 ratio	**0.50**	**0.0062**	0.13	0.50	**0.58**	**0.0012**	0.17	0.38

Virological biomarkers								
US RNA	−0.14	0.50	0.11	0.60	−0.09	0.65	0.14	0.48
MS RNA	0.36	0.068	**0.59**	**0.0011**	0.28	0.16	**0.44**	**0.023**
Total DNA	−0.07	0.74	−0.15	0.45	−0.02	0.92	−0.11	0.58
US RNA/total DNA ratio	0.00	0.99	0.10	0.63	0.01	0.95	0.14	0.50
US RNA/MS RNA ratio	**−0.53**	**0.0064**	**−0.62**	**0.0011**	**−0.56**	**0.0037**	**−0.55**	**0.0041**

CD4^+^ T-cell subsets								
CD4^+^ T_n_	**0.54**	**0.0056**	0.10	0.62	0.35	0.086	−0.09	0.67
CD4^+^ T_td_	0.04	0.85	0.19	0.37	0.03	0.90	0.21	0.30
CD4^+^ T_cm_	0.04	0.85	0.03	0.88	−0.10	0.61	−0.22	0.27
CD4^+^ T_tm_	−0.27	0.16	−0.07	0.71	−0.06	0.75	0.20	0.32
CD4^+^ T_em_	−0.37	0.056	−0.07	0.72	−0.26	0.18	0.05	0.80
CD4^+^/CD31^+^/CD45RA^+^	**0.58**	**0.0013**	0.14	0.47	0.31	0.11	−0.10	0.61
CD4^+^ naive T-cell CD31^+^ subset	**0.38**	**0.044**	0.08	0.69	−0.01	0.98	−0.28	0.15
CD4^+^/Ki67^+^	0.19	0.33	−0.01	0.95	0.16	0.40	−0.07	0.74
T_reg_	−0.31	0.12	−0.24	0.22	−0.27	0.17	−0.22	0.28

CD8^+^ T-cell subsets								
CD8^+^ T_n_	**0.55**	**0.0023**	0.18	0.37	**0.42**	**0.024**	0.02	0.90
CD8^+^ T_eff_	−0.33	0.089	−0.05	0.80	**−0.40**	**0.037**	0.00	0.98
CD8^+^ T_cm_	0.01	0.97	−0.03	0.86	−0.01	0.94	−0.22	0.26
CD8^+^ T_tm_	0.17	0.40	0.10	0.60	0.34	0.079	0.14	0.48
CD8^+^ T_em_	−0.19	0.32	−0.21	0.29	−0.09	0.63	−0.05	0.82
CD8^+^/Ki67^+^	0.00	0.98	0.22	0.26	−0.17	0.38	0.02	0.92

CD4^+^ activation and exhaustion								
CD4^+^/CD38^+^	0.19	0.33	0.01	0.95	−0.14	0.49	−0.35	0.067
CD4^+^/CD57^+^	−0.19	0.35	−0.05	0.80	−0.17	0.40	0.01	0.98
CD4^+^/CTLA-4^+^	0.00	0.99	0.18	0.35	0.07	0.71	0.18	0.36
CD4^+^/HLA-DR^+^	**−0.41**	**0.032**	−0.18	0.36	**−0.51**	**0.0055**	−0.29	0.13
CD4^+^/PD-1^+^	−0.28	0.15	−0.22	0.27	−0.32	0.095	−0.23	0.23
CD4^+^/HLA-DR^+^/CD38^+^	**−0.41**	**0.028**	**−0.39**	**0.042**	**−0.56**	**0.0021**	**−0.50**	**0.0065**
CD4^+^/CTLA-4^+^/PD-1^+^	−0.35	0.066	−0.19	0.34	−0.26	0.18	−0.15	0.45
CD4^+^/CD57^+^/PD-1^+^	−0.22	0.28	−0.12	0.54	−0.15	0.44	−0.05	0.79
CD4^+^/CD57^+^/HLA-DR^+^	−0.26	0.19	−0.07	0.74	−0.36	0.067	−0.16	0.43

CD8^+^ activation and exhaustion								
CD8^+^/CD38^+^	−0.08	0.69	0.06	0.77	−0.34	0.077	−0.20	0.31
CD8^+^/CD57^+^	−0.14	0.47	−0.12	0.54	−0.16	0.42	−0.08	0.70
CD8^+^/CTLA-4^+^	0.17	0.39	**0.41**	**0.029**	0.11	0.59	0.25	0.20
CD8^+^/HLA-DR^+^	−0.22	0.25	**−0.39**	**0.039**	−0.34	0.072	**−0.49**	**0.0075**
CD8^+^/PD-1^+^	0.32	0.096	0.05	0.81	−0.01	0.95	−0.29	0.13
CD8^+^/HLA-DR^+^/CD38^+^	−0.29	0.14	**−0.39**	**0.041**	**−0.45**	**0.016**	**−0.51**	**0.0058**
CD8^+^/CTLA-4^+^/PD-1^+^	0.21	0.28	0.33	0.089	0.07	0.71	0.10	0.62
CD8^+^/CD57^+^/PD-1^+^	0.12	0.56	0.05	0.79	−0.05	0.82	−0.14	0.49
CD8^+^/CD57^+^/HLA-DR^+^	−0.04	0.86	−0.10	0.63	−0.19	0.35	−0.18	0.36

CD4^+^ apoptosis								
CD4^+^/annexin V^+^	−0.27	0.18	0.00	1.00	−0.18	0.37	0.09	0.66
CD4^+^ Fas^+^	−0.34	0.081	−0.29	0.14	**−0.39**	**0.042**	−0.34	0.080
CD4^+^/annexin V^+^/Fas^+^	−0.38	0.051	−0.25	0.21	**−0.41**	**0.032**	−0.27	0.17
CD4^+^/annexin V^+^/CD38^+^	−0.15	0.47	−0.15	0.46	−0.30	0.12	−0.25	0.21
CD4^+^/annexin V^+^/HLA-DR^+^	**−0.41**	**0.032**	−0.14	0.49	**−0.40**	**0.040**	−0.12	0.55
CD8^+^ apoptosis								
CD8^+^/annexin V^+^	−0.20	0.31	−0.07	0.72	−0.07	0.73	0.08	0.70
CD8^+^ Fas^+^	−0.20	0.31	−0.26	0.18	−0.36	0.069	**−0.44**	**0.023**
CD8^+^/annexin V^+^/Fas^+^	−0.18	0.38	−0.24	0.24	−0.34	0.079	**−0.42**	**0.027**
CD8^+^/annexin V^+^/CD38^+^	−0.35	0.073	−0.16	0.44	−0.36	0.063	−0.19	0.34
CD8^+^/annexin V^+^/HLA-DR^+^	−0.24	0.24	−0.27	0.17	−0.23	0.25	−0.29	0.15

a Significant *P* values are shown in boldface type, and those that remained significant after Benjamini-Hochberg correction for multiple comparisons are underlined.

Next, we assessed the predictive value of the 12-week biomarkers for the absolute and relative CD4^+^ counts at 48 and 96 weeks of ART by multivariable modeling. Only those markers that were significantly associated with the endpoints after correction for multiple comparisons were included in the models (Table 3). In the multivariable analysis, the US/MS RNA ratio remained significantly predictive of three out of four endpoints: the absolute CD4^+^ count at week 48 (regression coefficient [B] = −12.9 [95% CI, −19.9 to −5.98]; *P* = 2.7 × 10^−4^), the relative CD4^+^ count at week 48 (B = −14.2 [−24.3 to −4.1]; *P* = 0.0060), and the relative CD4^+^ count at week 96 (B = −12.6 [−21.3 to −3.99]; *P* = 0.0042). For the absolute CD4^+^ count at week 96, a trend was observed (B = −6.48 [−14.6 to 1.67]; *P* = 0.12). Other predictors that remained significant in the multivariable analysis were the CD4^+^ count and CD8^+^ T_n_ cells for the absolute CD4^+^ count at week 48, the CD4/CD8 ratio for the absolute CD4^+^ count at week 96, and CD8^+^/CD38^+^/HLA-DR^+^ cells for the relative CD4^+^ count at week 96. As a sensitivity analysis, we also built bivariable models in which we included only the US/MS RNA ratio and the CD4^+^/CD38^+^/HLA-DR^+^ cell percentage at 12 weeks of ART, as these markers were strongly correlated, and both of them were predictive of the immunological response. The results of this analysis (see the supplemental table available at https://doi.org/10.6084/m9.figshare.13523948) were similar to those of the multivariable analysis presented above (Table 3): for the same three out of four endpoints, the US/MS RNA ratio remained significantly predictive also after adjustment for the percentage of CD4^+^ cells that were HLA-DR^+^/CD38^+^, whereas for the absolute CD4^+^ count at week 96, a trend was observed (B = −8.98 [−19.6 to 1.66]; *P* = 0.098). In contrast, the CD4^+^/CD38^+^/HLA-DR^+^ cell percentage was not significantly predictive of any endpoint when adjusted for the US/MS RNA ratio. Therefore, the conclusions remained unchanged.

**TABLE 3:** Multivariable analysis of the predictive value of biomarkers measured at 12 weeks of ART for the absolute and relative CD4^+^ counts at 48 and 96 weeks of ART

Dependent variable and predictive biomarker^*a*^	B (95% CI)	*P*
Absolute CD4^+^ count at 48 wks of ART		
CD4^+^ count, per cell/mm^3^	0.53 (0.20 to 0.87)	0.0019
CD4/CD8 ratio, per 0.1	−24.1 (−58.5 to 10.3)	0.17
US RNA/MS RNA ratio, per 0.1 log_10_	−12.9 (−19.9 to −5.98)	2.7 × 10^−4^
CD4^+^ T_n_, per %	0.20 (−5.69 to 6.09)	0.95
CD4^+^/CD31^+^/CD45RA^+^, per %	−0.93 (−8.16 to 6.29)	0.80
CD8^+^ T_n_, per %	9.82 (0.74 to 18.9)	0.034

Relative CD4^+^ count at 48 wks of ART		
MS RNA, per 0.1 log_10_	2.37 (−6.40 to 11.1)	0.60
US RNA/MS RNA ratio, per 0.1 log_10_	−14.2 (−24.3 to −4.1)	0.0060
	
Absolute CD4^+^ count at 96 wks of ART		
CD4^+^ count, per cell/mm^3^	0.18 (−0.16 to 0.52)	0.30
CD4/CD8 ratio, per 0.1	24.1 (6.29 to 41.9)	0.0080
US RNA/MS RNA ratio, per 0.1 log_10_	−6.48 (−14.6 to 1.67)	0.12
CD4^+^/HLA-DR^+^, per %	10.2 (−2.34 to 22.2)	0.11
CD4^+^/HLA-DR^+^/CD38^+^, per %	−18.6 (−43.4 to 6.34)	0.14

Relative CD4^+^ count at 96 wks of ART		
US RNA/MS RNA ratio, per 0.1 log_10_	−12.6 (−21.3 to −3.99)	0.0042
CD4^+^/HLA-DR^+^/CD38^+^, per %	6.69 (−5.73 to 19.1)	0.29
CD8^+^/HLA-DR^+^, per %	12.1 (−2.25 to 26.4)	0.099
CD8^+^/HLA-DR^+^/CD38^+^, per %	−37.4 (−64.1 to −10.7)	0.0060

a Biomarkers that were significantly associated with the endpoints after correction for multiple comparisons (Table 2) were included in the models.

Finally, we assessed the correlations of the US/MS RNA ratio with other biomarkers at 12 weeks of ART after correction for multiple comparisons. Although the US/MS RNA ratio positively correlated with a number of markers of CD4^+^ T-cell activation and apoptosis, only correlations with two markers of activation (CD4^+^/HLA-DR^+^ and CD4^+^/CD38^+^/HLA-DR^+^) and two markers of apoptosis (CD4^+^/annexin V^+^/Fas^+^ and CD4^+^/annexin V^+^/HLA-DR^+^) remained significant (Table S4).

10.1128/mBio.00099-21.9 TABLE S4 Immunological biomarkers associated with the US RNA/MS RNA ratio at 12 weeks of ART. Download Table S4, PDF file, 0.03 MB. Copyright © 2021 Scherpenisse et al. 2021 Scherpenisse et al. https://creativecommons.org/licenses/by/4.0/ This content is distributed under the terms of the Creative Commons Attribution 4.0 International license.

## DISCUSSION

In this study, we longitudinally measured a large number of virological and immunological biomarkers at baseline and throughout the first 96 weeks of suppressive ART, determined their dynamics and mutual correlations, and assessed their predictive value for the degree of immunological response to therapy. To the best of our knowledge, this is by far the largest such study in terms of the number of measured biomarkers and the intensity of the measurements. Previous studies of the immunological response to ART were mostly cross-sectional and therefore could identify only correlates but not predictors of immune recovery, and those studies that were longitudinal measured fewer biomarkers.

As expected, the levels of all virological biomarkers decreased after ART initiation. However, the longitudinal dynamics of total HIV-1 DNA and CA RNA were strikingly different. While total HIV-1 DNA gradually decreased by ∼10-fold within the first 96 weeks of ART, CA US and MS RNAs dropped already by ∼10-fold and >100-fold, respectively, within the first 12 weeks and remained relatively stable afterward. At every time point, the relative decreases of HIV-1 DNA from the baseline were the smallest, followed by US RNA, and MS RNA demonstrated the largest decreases. Similar differences in the dynamics of HIV-1 DNA and CA RNA decay on ART were reported previously (47, 83–87). The gradual decrease in HIV-1 DNA could reflect the elimination of rare cells harboring intact proviruses by the host immune response in the presence of the large background of defective proviruses that do not decay on therapy (88, 89). On the other hand, the biphasic kinetics of CA RNA reflects the steep decline of productively infected cells upon ART initiation (90), followed by a quasi-steady state that is fueled by stochastic reactivation of latently infected cells. The fact that MS RNA decreases on ART faster and plateaus at a much lower level than US RNA has also been observed previously (84, 91) and may reflect additional latency blocks to the activation of MS RNA expression compared with US RNA (92). It may also indicate (Tat-independent) transcription from defective proviruses with intact *gag* sequences but with deletions in the *tat*-*rev* region, splice sites, or exonic splicing enhancers.

Prominent changes on ART were observed in the levels of markers of immune activation and apoptosis. These markers demonstrated a steady decrease from pre-ART levels, but most of them still did not achieve the levels of HDs within the first 96 weeks of ART, in agreement with previous reports (15, 24). Partial or full normalization was also observed in some (but not all) T-cell subsets, similarly to a previous report by Breton et al. (93). In contrast, most markers of immune proliferation, senescence, and exhaustion did not change from pre-ART levels, indicating the persistence of residual immune senescence and dysfunction during at least the first 96 weeks of ART (33, 80, 94).

To our knowledge, this is the first study to demonstrate that a cell-associated HIV-1 marker is predictive of the immunological response to ART. Because of the large number of biomarkers measured in this study, a stringent correction for multiple comparisons was necessary, but the US/MS RNA ratio remained significantly predictive of all four endpoints after correction for multiple comparisons and of three of these endpoints in the subsequent multivariable analysis. These results, coupled to our previous observations that CA HIV-1 US RNA predicts virological failure on ART, correlates with small nonadherence to therapy, and predicts the time and magnitude of viral rebound after ART interruption (47, 95, 96), position CA HIV-1 RNA as a major virological biomarker that is strongly predictive of a number of different clinical endpoints.

Remarkably, the US/MS RNA ratio, which was negatively predictive of the immunological response to ART in this study, was shown by several groups in the 1990s to be associated with disease progression in untreated individuals. Higher US/MS RNA ratios were measured in typical/rapid progressors, while slow progressors and long-term nonprogressors were characterized by low ratios (97–101). Several possible explanations can be put forward for these associations of the US/MS RNA ratio with rapid progression in untreated individuals and with immunological failure on ART. First, the HIV-1 replication cycle involves a temporal shift from the production of MS to the production of US RNA, as observed both in *in vitro* HIV-1 infection (102) and after stimulation of latently infected cell lines (103–105). Therefore, a higher US/MS RNA ratio in an infected individual might reflect a higher frequency of HIV-infected cells in the later stages of the viral replication cycle, which is characterized by elevated expression of viral proteins and production of virus particles. Such cells could exert pressure on the host immune system, causing persistent immune activation and apoptosis and thus contributing to rapid disease progression and a poor immunological response to ART. Indeed, the US/MS RNA ratio was strongly associated with markers of CD4^+^ T-cell activation and apoptosis in this study. Second, for untreated infection, it was proposed that weaker anti-HIV cytotoxic T lymphocyte (CTL) responses would lead to cells in the late, productive phase of infection being killed at a reduced rate, resulting in the preponderance of cells with increased US/MS RNA ratios (106). In line with this, rapid progression was shown to be associated both with weaker CTL responses and higher US/MS RNA ratios (98). The same may be true for the antiviral immune response during ART. In fact, the CTL response has been proposed as the major mechanism behind the selective elimination of intact, HIV-1 RNA-expressing proviruses on therapy (88, 89). Third, Kaiser et al. measured the US and MS RNA levels in resting and activated CD4^+^ T cells from the same ART-treated individuals (107). From their report, it can be derived that US/MS RNA ratios in resting CD4^+^ cells are on average 1:1, whereas these ratios in activated CD4^+^ cells are 27:1. Activated CD4^+^ cells are more permissive for the later stages of HIV-1 replication cycle than resting cells (108, 109), and thus, a high US/MS RNA ratio may reflect the relative abundance of (re)activated, compared to resting, HIV-infected CD4^+^ cells. Resting memory CD4^+^ T cells are the major HIV-1 reservoir during ART (110, 111), but resting cells are constantly being reactivated in response to their cognate antigens or to cytokines. In turn, the frequency of reactivation could reflect the level of CD4^+^ T-cell activation in an individual. Indeed, in this report, the CD4^+^ T-cell activation level correlated positively with US/MS ratios and negatively with CD4^+^ counts on ART. Furthermore, higher levels of CD4^+^ T-cell activation at 12 weeks of ART predicted poorer immune reconstitution, although its predictive value was no longer significant in the multivariable analysis. To summarize, our results contribute to the view that persistence of cells with higher US/MS RNA ratios (and, in general, HIV-1 persistence on ART) could be both a cause and a consequence of increased immune activation. Both viral and host factors are likely interdependent in this process and, in concert, contribute to the poor immunological response to ART.

One intriguing aspect of this study is the difference between baseline markers and the same markers measured at 12 weeks of ART for the prediction of immune reconstitution. Some baseline markers, such as plasma viral load, MS RNA, RTEs, or CTLA-4 expression on CD4^+^ and CD8^+^ T cells, were associated with one or more endpoints, but the correlations were modest, and the significance was lost after correction for multiple comparisons. Age and ART regimen did not demonstrate a significant predictive value for immune reconstitution either. ART regimens were shown previously not to contribute to immunological failure as long as ART is suppressive (34), and most participants in this study were on protease inhibitor-based triple-ART regimens for the duration of the follow-up. However, older age is known to contribute to poor immune reconstitution; therefore, the lack of an effect of age in this study is surprising and can possibly be explained by the limited number of participants and their narrow age range.

In contrast, a number of markers measured at 12 weeks were strongly predictive of immune reconstitution. HIV-1 biology, as well as the state of host immunity, is very different between untreated and treated infections. Therefore, levels of biomarkers measured in untreated infection may bear relatively little significance for subsequent immune restoration on ART, while the state of the virus and host after ART has been initiated is likely more relevant. Interestingly, positive correlations between the viral and host biomarkers at 12 weeks of ART were stronger than at baseline or at subsequent on-ART time points. In particular, US RNA/total DNA and US/MS RNA ratios, representing relative HIV-1 transcriptional activity per provirus and the relative number of cells in the later stages of productive infection, respectively, strongly correlated with multiple markers of immune activation, apoptosis, and exhaustion at 12 weeks of ART but not before or after that time point.

Why 12 weeks of ART is “special” in this respect is unclear, but one possible explanation is that a distinct population of infected cells predominates early on ART. The decay of HIV-infected cells after ART initiation is multiphasic (90, 112, 113), with short-lived cells dominating the total infected cell pool during the first months of ART. Rosenbloom et al. estimated that at 3 months of ART, >90% of infected cells are labile, masking the persistent viral reservoir (114). The nature of these cells, which decay out during the first year of therapy, is still unclear, but their biology and, hence, the state of HIV-1 infection of these cells are likely different from those of the long-lived reservoir cells that support latent infection. It is possible that the activation level of these cells is higher and therefore they impose fewer blocks to productive HIV-1 infection than the long-lived reservoir cells. Hence, the US/MS RNA ratio in such cells can signify the relative number of productively infected cells that, as discussed above, either directly influences the subsequent immunological response to ART or reflects the impaired state of host immunity that contributes to immune failure. In contrast, later on ART, when most HIV-1 is found in long-lived reservoir cells that either are latently infected or harbor defective proviruses, a higher US/MS RNA ratio may represent the opposite: a higher relative number of cells with posttranscriptional latency blocks that prevent efficient completion of transcription and splicing (92). The US/MS RNA ratio and most other HIV-1 markers in such cells are not expected to correlate with the state of host immunity, and indeed, Gandhi et al., who measured the associations between virological and immunological markers at years 1 and 4 of ART but not earlier, did not find any correlations (45). Similarly, Spudich et al. did not find any correlations between inflammatory biomarkers in the cerebrospinal fluid (CSF) and HIV persistence measures in a cohort of ART-treated individuals with a median of 8.6 years on therapy, although total HIV-1 DNA detectability in CSF was associated with worse neurocognitive outcomes (46). However, other groups found some positive correlations between HIV-1 DNA and CA RNA and markers of immune activation in individuals on ART (30, 42). To our knowledge, no group previously assessed correlations between US RNA/total DNA and US/MS RNA ratios and host markers.

Our study has some limitations. First, we included a limited number of participants. However, in this cohort, we performed a very detailed analysis, longitudinally measuring more than 50 biomarkers at five time points during ART. Second, most participants started ART more than 20 years ago, meaning that during the study period, they were treated with ART regimens that are currently not recommended for first-line therapy and that their virological response to therapy was measured by plasma viral load assays with higher detection limits (mostly 400 copies/ml) than the ones currently used. However, participants were always at least on triple ART, and all of them achieved virological suppression by 24 weeks of ART (all except two participants achieved virological suppression already by 12 weeks of ART) and maintained it throughout the study period. Although one is unable to know the exact degree of virological suppression in an individual with a plasma viral load of <400 copies/ml, and the possibility of intermittent low-level HIV-1 replication in some participants cannot be entirely excluded, only 4 out of 28 participants demonstrated blips after achieving virological suppression. Moreover, we observed substantial increases in CD4^+^ counts and CD4/CD8 ratios on ART, and a number of immunological markers partly or fully normalized on therapy, indicating that ART in this study was indeed suppressive. Also, the longitudinal dynamics of CA HIV-1 RNA and total HIV-1 DNA after ART initiation in this cohort were virtually indistinguishable from those measured in another, more recent cohort treated with newer ART regimens and with plasma viral loads quantified using more sensitive assays (115). Further research is warranted in order to establish the predictive role of the US/MS RNA ratio for the immunological response to modern ART regimens.

Although several correlates of a poor immunological response such as lower naive T-cell percentages or higher levels of immune activation have been previously reported, the HIV-1 research field and clinical care are still lacking reliable biomarkers that could predict immunological failure. The main novelty and strength of this study are that we identified an HIV-1 marker that outperformed all other markers in the prediction of the immunological response to ART. The fact that a virological biomarker performed better than the immunological biomarkers in predicting an immunological outcome highlights the importance of considering the residual HIV-1 activity on suppressive ART as a correlate, and a possible cause, not only of the poor immune reconstitution on therapy but also of the residual immune dysfunction that frequently occurs despite virologically suppressive ART (33, 116). In this regard, recently identified drugs that target HIV-1 transcription (117–120) may have the potential to suppress immune activation and dysfunction and facilitate immune reconstitution on ART. In addition, our results suggest that an early time point after ART initiation could be more informative than the baseline for assessing the impact of residual HIV-1 activity on the subsequent immunological response. Further studies in larger cohorts are necessary to fully understand the impact of HIV-1 persistence on immune reconstitution on ART.

## MATERIALS AND METHODS

### Participants and samples.

We used archival PBMC samples from HIV-infected individuals who participated in the Amsterdam Cohort Studies (ACS) on HIV infection and AIDS. To select participants for this study, we performed extensive screening of the ACS sample collection. Out of 483 individuals treated with combination ART, we selected 28 participants who started ART with CD4^+^ counts of <350 cells/mm^3^ and plasma viral loads of >1,000 copies/ml, who achieved durable virological suppression on ART (suppressed plasma viral loads by 24 weeks of ART and thereafter up to 96 weeks, allowing blips of <1,000 copies/ml), and for whom at least four longitudinal PBMC samples, corresponding to five time points (0, 12, 24, 48, and 96 weeks) on ART, were available. The median age of participants at the start of ART was 39 years, and all were males. Participants were treated with combination ART that started, on average, in May 1997 and initially consisted of two nucleoside reverse transcriptase inhibitors and at least one protease inhibitor. By week 96 of ART, a small percentage of participants switched to a nonnucleoside reverse transcriptase inhibitor-based ART regimen.

For 25 participants, PBMC samples at all five time points were available, and for 3 participants, a sample at 24 weeks of ART was missing. In total, 137 PBMC samples were included in the analysis. For the baseline measurements, 25 samples were obtained immediately prior to the start of ART, and 3 samples were obtained a median of 2 weeks before ART initiation. For the 12-week measurements, samples were obtained at a median of 1 week (IQR,1 to 2 weeks) before or after the exact 12-week time point. For the 24-week, 48-week, and 96-week measurements, these differences were 2 (interquartile range, 0 to 4) weeks, 3 (2 to 5) weeks, and 4 (2 to 8) weeks, respectively.

PBMCs of HDs (*n* = 6) were obtained from the Sanquin blood bank. No specific information about the HDs was available.

### Quantification of virological biomarkers.

Plasma viral load was measured using commercial assays with detection limits of 1,000, 400, or 50 copies/ml. For CA HIV-1 RNA and DNA measurements, total nucleic acids were extracted from PBMCs using the Boom isolation method (121). Extracted cellular RNA was treated with DNase (DNA-free kit; Thermo Fisher Scientific) to remove DNA that could interfere with quantitation and reverse transcribed using random primers and SuperScript III reverse transcriptase (all from Thermo Fisher Scientific). CA HIV-1 US RNA and total HIV-1 DNA were measured using previously described seminested quantitative PCR (qPCR)-based assays (122). CA HIV-1 MS RNA was measured using a novel seminested qPCR-based assay that quantifies the cumulative copy numbers of MS RNA species of *tat*, *rev*, and *nef* genes. The first PCR was performed using oligonucleotide primers MS_total (5′-GAAGAAGCGGAGACAGCGACGA-3′) and mf83, and qPCR was performed with primers mf84 and mf83 and probe ks2-tq (122). Apart from a different forward primer for the first PCR, the MS RNA assay was performed essentially as previously described (122). HIV-1 DNA or RNA copy numbers were determined using a 7-point standard curve with a linear range of more than 5 orders of magnitude that was included in every qPCR run and normalized to the total cellular DNA (by measurement of β-actin DNA) or RNA (by measurement of 18S rRNA) inputs, respectively, as described previously (47). Quantification standards for total HIV-1 DNA and CA US and MS HIV-1 RNAs were described previously (122).

At baseline, both total HIV-1 DNA and US RNA were undetectable in one participant, who was therefore presumed to be infected with a non-B HIV-1 subtype, and all his HIV-1 DNA and US RNA measurements were excluded from the analysis. Of the on-ART samples, total DNA was detectable in 100%, US RNA was detectable in 98%, and MS RNA was detectable in 76%. Undetectable measurements of CA RNA were assigned values corresponding to the assay detection limits, with a maximum of 100 copies/μg total cellular RNA. The detection limits depended on the amounts of the normalizer (input cellular RNA) and therefore differed between samples. Measurements with low input cellular RNA and undetectable HIV-1 RNA (US RNA, *n* = 3; MS RNA, *n* = 2) were excluded from the analysis. As a sensitivity analysis, we either assigned values corresponding to 50%, instead of 100%, of the detection limits to the undetectable samples or excluded the undetectable samples completely from the analysis. In both cases, the conclusions of the study were unaffected (data not shown).

### Quantification of immunological biomarkers.

PBMCs from HIV-infected participants and HDs were washed and stained with antibodies or annexin V for 30 min in the dark to determine the expression of immunological biomarkers on CD4^+^ and CD8^+^ T cells or their apoptotic state. Four eight-color flow cytometry panels were used. Panel 1 contained CD3-Alexa Fluor 700 (clone HIT3a, catalog number 300324; BioLegend), CD4-fluorescein isothiocyanate (FITC) (clone RPA-T4, catalog number 300506; BioLegend), CD8-allophycocyanin (APC)-H7 (clone SK1, catalog number 561423; BD), CD45RA-phycoerythrin (PE)-Cy7 (clone L48, catalog number 337186; BD), CCR7-PE-CF594 (clone 150503, catalog number 562381; BD), CD27-peridinin chlorophyll protein (PerCP)-Cy5.5 (clone M-T271, catalog number 356408; BioLegend), CD31-PE (clone WM59, catalog number 303106; BioLegend), and Ki67-APC (clone Ki-67, catalog number 350514; BioLegend). Panel 2 contained CD3-Alexa Fluor 700 (clone HIT3a, catalog number 300324; BioLegend), CD4-FITC (clone RPA-T4, catalog number 300506; BioLegend), CD8-APC-H7 (clone SK1, catalog number 561423; BD), CD38-PE-CF594 (clone HIT2, catalog number 562288; BD), HLA-DR–PE–Cy7 (clone G46-6, catalog number 560651; BD), CD57-PE (clone HNK-1, catalog number 359612; BioLegend), PD-1–PerCP–Cy5.5 (clone EH12.1, catalog number 561273; BD), and CTLA-4–APC (clone L3D10, catalog number 349907; BioLegend). Panel 3 contained CD3-Alexa Fluor 700 (clone HIT3a, catalog number 300324; BioLegend), CD4-FITC (clone RPA-T4, catalog number 300506; BioLegend), CD25-PE (clone M-A251, catalog number 555432; BD), and FoxP3-PerCP-Cy5.5 (clone 236A/E7, catalog number 561493; BD). Panel 4 contained CD3-Alexa Fluor 700 (clone HIT3a, catalog number 300324; BioLegend), CD4-FITC (clone RPA-T4, catalog number 300506; BioLegend), CD8-APC-H7 (clone SK1, catalog number 561423; BD), Fas-PE (clone DX2, catalog number 340480; BD), HLA-DR–PE–Cy7 (clone G46-6, catalog number 560561; BD), CD38-PE-CF594 (clone HIT2, catalog number 562288; BD), and annexin V-APC (catalog number 550474; BD). Antibody dilution factors are shown in Table S5 in the supplemental material. Examples of the gating strategy are shown in the supplemental figure available at https://doi.org/10.6084/m9.figshare.12942860. Gates were defined using fluorescence-minus-one controls. Fluorescence was measured with the BD FACSCanto II cell analyzer. Analyses were performed with the FlowJo V10 analysis platform.

10.1128/mBio.00099-21.10 TABLE S5 Antibody panels to measure the expression of immunological biomarkers. Download Table S5, PDF file, 0.01 MB. Copyright © 2021 Scherpenisse et al. 2021 Scherpenisse et al. https://creativecommons.org/licenses/by/4.0/ This content is distributed under the terms of the Creative Commons Attribution 4.0 International license.

### Statistical analysis.

Longitudinal dynamics of virological and immunological biomarkers were modeled using repeated-measures mixed-effects analysis with Tukey corrections for multiple comparisons. The Greenhouse-Geisser correction was applied to adjust for the lack of sphericity. Mann-Whitney tests were used to compare the 96-week values of biomarkers with those of HDs. Pairwise correlations between biomarkers per time point and between time points per biomarker were determined by Spearman tests. Correlations of biomarkers with absolute and relative CD4^+^ counts and correlations of US/MS RNA ratios with other biomarkers were determined by Spearman tests with Benjamini-Hochberg corrections for multiple comparisons (false discovery rate, 0.1). Kruskal-Wallis tests with Dunn’s posttests were used to compare the immunological responses between participants treated with different ART regimens. Immunological responses were compared between individuals with high and those with low US/MS RNA ratios at 12 weeks of ART using Mann-Whitney tests. Multivariable analyses of biomarkers predicting the immunological response were performed by fitting generalized linear models (GLMs). Data were analyzed using Prism 8.3.0 (GraphPad Software) or IBM SPSS Statistics 25. All statistical tests were two sided, and *P* values of <0.05 were considered statistically significant.

### Ethics statement.

The study has been approved by the ACS committee. The ACS have been approved by the Medical Ethical Committee of the Academic Medical Center (approval number MEC 07/182). The ACS have been conducted in accordance with the ethical principles set out in the Declaration of Helsinki, and written informed content was obtained prior to sample collection.
