# Supplementary material for: Cell-Associated HIV-1 Unspliced-to-Multiply-Spliced RNA Ratio at 12 Weeks of ART Predicts Immune Reconstitution on Therapy
Source: mBio. 2021 Mar 9;12(2):e00099-21. doi: 10.1128/mBio.00099-21 (PMC8092199; doi:10.1128/mBio.00099-21)
Supplement: FIG S1 [file mBio.00099-21-sf001.pdf]

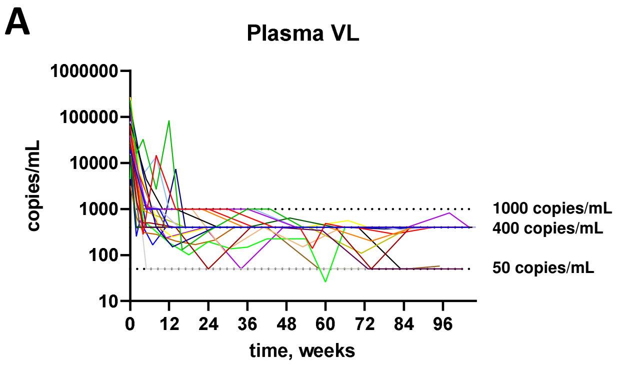

Figure S1. Longitudinal changes of plasma viral load (VL) and CD4+ count during the first 96 weeks of ART. Lines correspond to individual participants and are color-coded. For plasma VL, limits of detection of the commercial assays are shown with dashed lines.
