# Supplementary material for: Cell-Associated HIV-1 Unspliced-to-Multiply-Spliced RNA Ratio at 12 Weeks of ART Predicts Immune Reconstitution on Therapy
Source: mBio. 2021 Mar 9;12(2):e00099-21. doi: 10.1128/mBio.00099-21 (PMC8092199; doi:10.1128/mBio.00099-21)
Supplement: TABLE S1 [file mBio.00099-21-st001.pdf]

Table S1. Baseline and treatment characteristics of the study participants (n=28).

Age, years		39 (35-45)[a]
Male gender		100 (28/28)
CD4+ count nadir, cells/mm^3		195 (148-250)
Treatment experience before the start of combination ART[b]		28.6 (8/28)
Baseline plasma viral load, $\log_{10}$ copies/ml		4.58 (4.35-4.86)
Baseline CD4+ count, cells/mm^3		260 (150-300)
Baseline CD4/CD8 ratio		0.22 (0.12-0.33)
Baseline cell-associated HIV unspliced RNA in PBMC, $\log_{10}$ copies/µg total RNA		4.24 (3.93-4.54)
Baseline cell-associated HIV multiply spliced RNA in PBMC, $\log_{10}$ copies/µg total RNA		3.30 (3.03-3.69)
Baseline total HIV DNA, $\log_{10}$ copies/10^6 PBMC		3.75 (3.61-4.27)
Calendar month of start ART, median (range)		05/1997 (07/1996-09/2002)
NRTI backbone at the start of ART	AZT+3TC	14.3 (4/28)
	D4T+3TC	46.4 (13/28)
	D4T+DDI	14.3 (4/28)
	unknown	25.0 (7/28)
PI at the start of ART	IDV	50.0 (14/28)
	NFV	7.1 (2/28)
	SQV	7.1 (2/28)
	SQV/r	7.1 (2/28)
	NFV+SQV	3.6 (1/28)
	unknown	25.0 (7/28)
NRTI backbone at 96 weeks of ART	AZT+3TC	17.9 (5/28)
	D4T+3TC	50.0 (14/28)
	D4T+DDI	17.9 (5/28)
	unknown	14.3 (4/28)
PI or NNRTI at 96 weeks of ART	IDV	50.0 (14/28)
	NFV	7.1 (2/28)
	SQV/r	10.7 (3/28)
	NVP	10.7 (3/28)
	NFV+SQV	3.6 (1/28)
	SQV/r+NVP	3.6 (1/28)
	NVP+urea	3.6 (1/28)
	unknown	10.7 (3/28)

[a] Data are medians (interquartile ranges) for continuous variables and % (proportions) for discrete variables, except where indicated.
[b] Mono- or dual-NRTI regimens.
