# Supplementary material for: Cell-Associated HIV-1 Unspliced-to-Multiply-Spliced RNA Ratio at 12 Weeks of ART Predicts Immune Reconstitution on Therapy
Source: mBio. 2021 Mar 9;12(2):e00099-21. doi: 10.1128/mBio.00099-21 (PMC8092199; doi:10.1128/mBio.00099-21)
Supplement: FIG S2 [file mBio.00099-21-sf002.pdf]

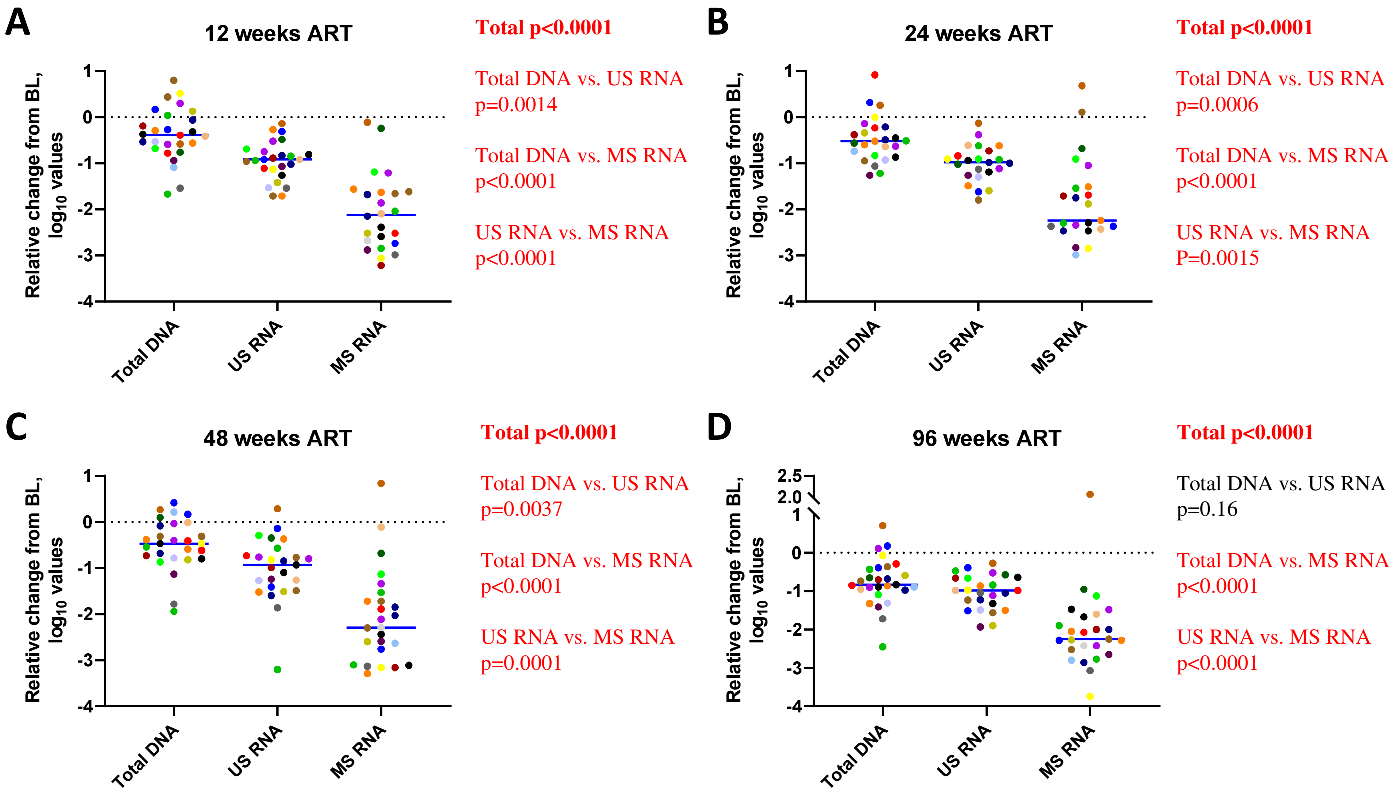

Figure S2. Comparisons of the relative changes of virological biomarkers from baseline at 12, 24, 48, and 96 weeks of ART. Participants are color-coded. Repeated measures mixed-effects p values as well as p values of pairwise comparisons between the biomarkers are depicted to the right of the corresponding graphs. Significant effects are shown in red.
