# Supplementary material for: Cell-Associated HIV-1 Unspliced-to-Multiply-Spliced RNA Ratio at 12 Weeks of ART Predicts Immune Reconstitution on Therapy
Source: mBio. 2021 Mar 9;12(2):e00099-21. doi: 10.1128/mBio.00099-21 (PMC8092199; doi:10.1128/mBio.00099-21)
Supplement: FIG S4 [file mBio.00099-21-sf004.pdf]

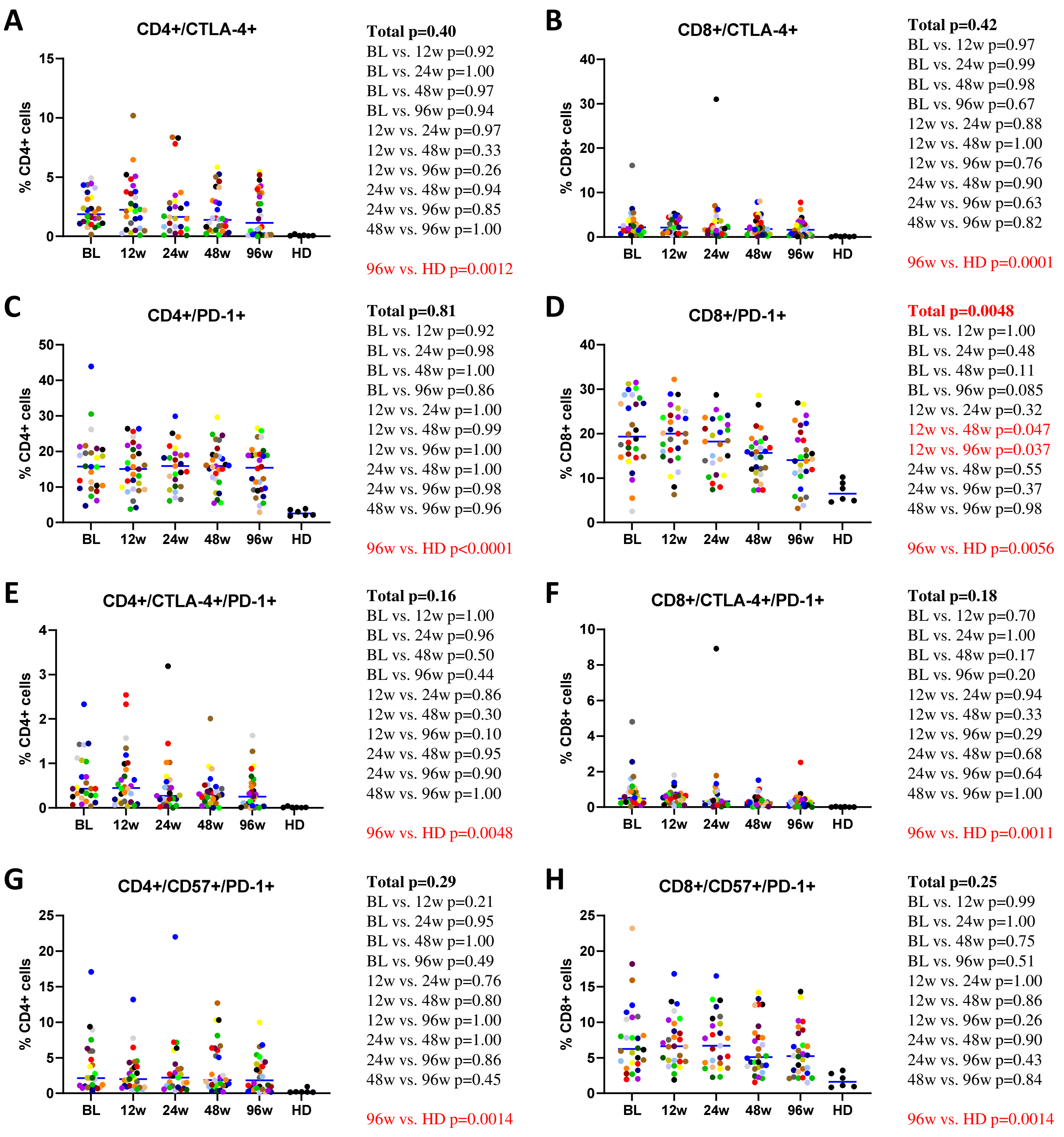

Figure S4. Longitudinal dynamics of markers of CD4+ and CD8+ exhaustion during the first 96 weeks of ART. Participants are color-coded. Repeated measures mixed-effects p values as well as p values of pairwise comparisons between the biomarker values at different time points and of comparisons between 96 week-values of HIV-infected participants and those of healthy donors (HD) are depicted to the right of the corresponding graphs. Significant effects are shown in red.
