# Supplementary material for: Cell-Associated HIV-1 Unspliced-to-Multiply-Spliced RNA Ratio at 12 Weeks of ART Predicts Immune Reconstitution on Therapy
Source: mBio. 2021 Mar 9;12(2):e00099-21. doi: 10.1128/mBio.00099-21 (PMC8092199; doi:10.1128/mBio.00099-21)
Supplement: FIG S5 [file mBio.00099-21-sf005.pdf]

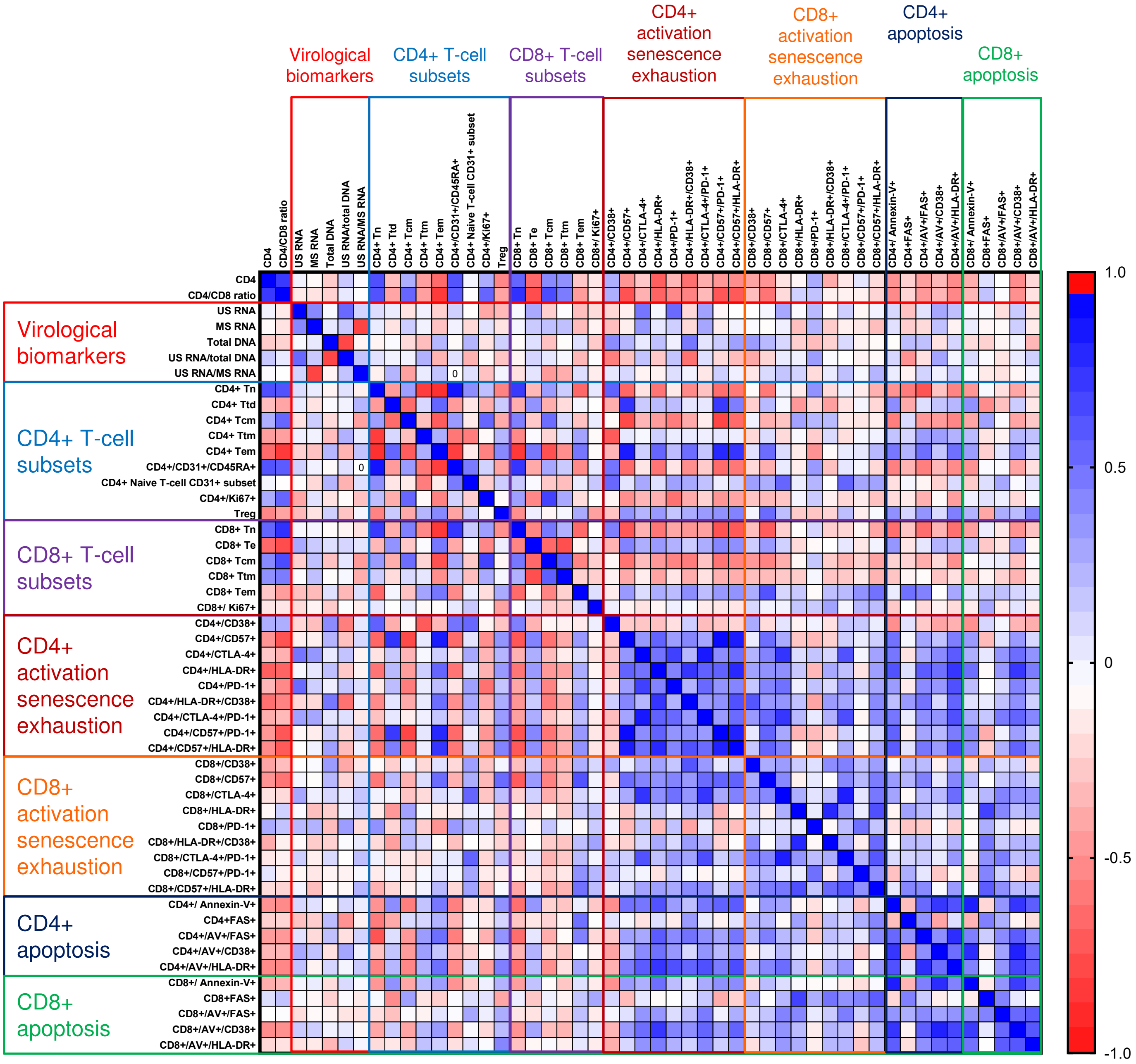

Figure S5A. Spearman correlogram of CD4+ count, CD4/CD8 ratio, virological and immunological biomarkers at 24 weeks of ART. Heat map is used to indicate the strengths of associations between biomarkers. Red indicates a negative correlation, blue a positive correlation.

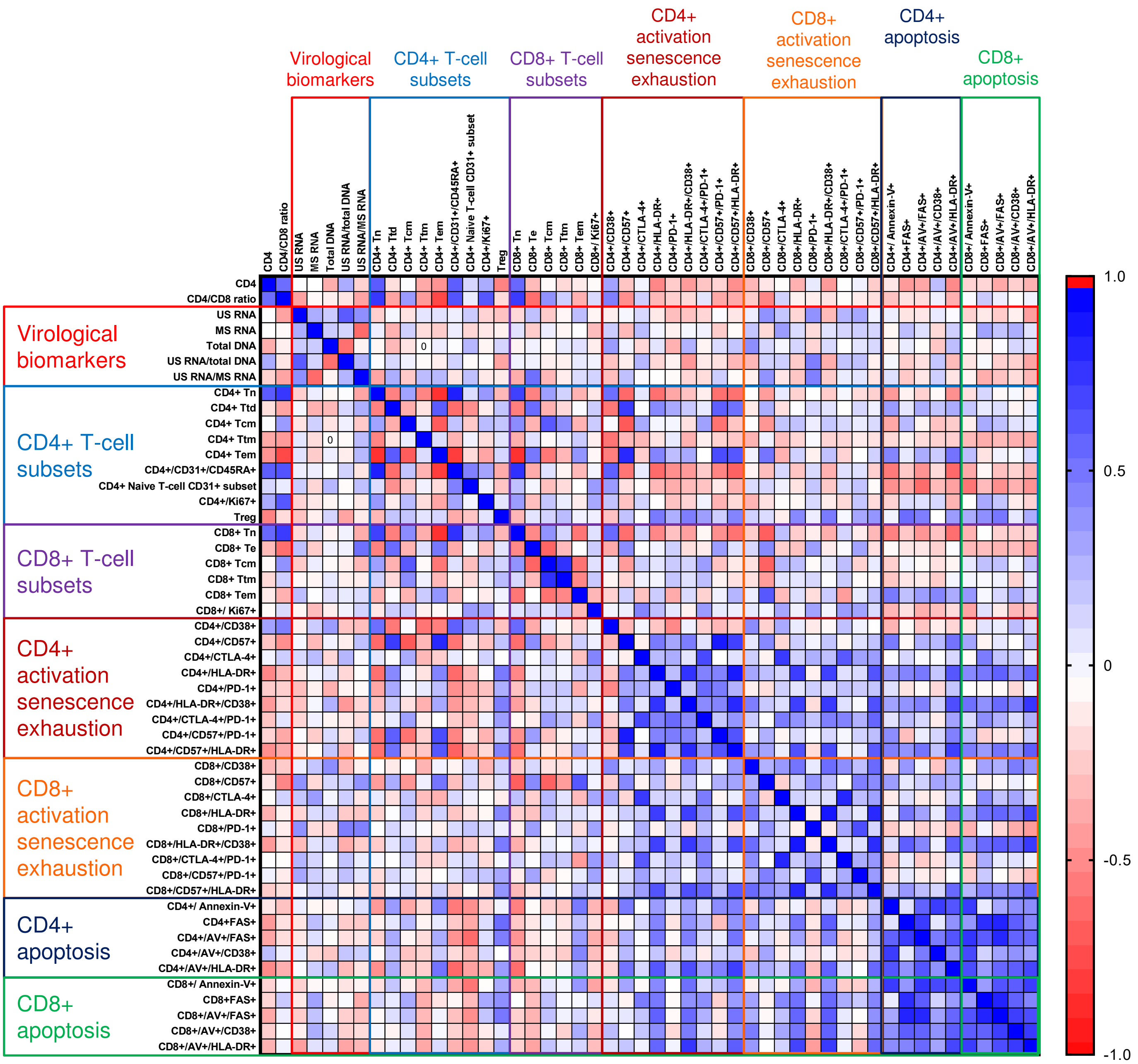

Figure S5B. Spearman correlogram of CD4+ count, CD4/CD8 ratio, virological and immunological biomarkers at 48 weeks of ART. Heat map is used to indicate the strengths of associations between biomarkers. Red indicates a negative correlation, blue a positive correlation.

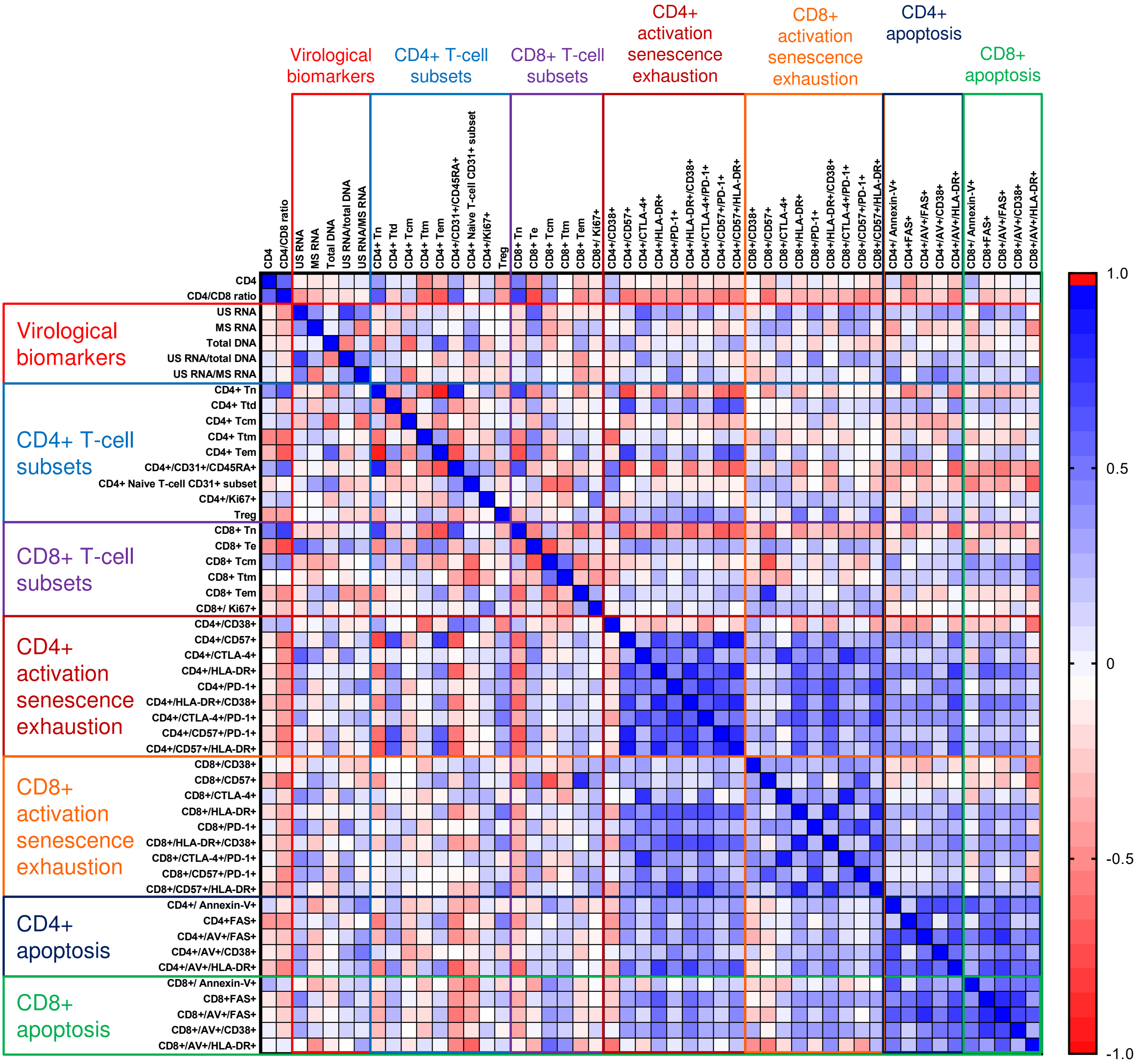

Figure S5C. Spearman correlogram of CD4+ count, CD4/CD8 ratio, virological and immunological biomarkers at 96 weeks of ART. Heat map is used to indicate the strengths of associations between biomarkers. Red indicates a negative correlation, blue a positive correlation.
