# Supplementary material for: Cell-Associated HIV-1 Unspliced-to-Multiply-Spliced RNA Ratio at 12 Weeks of ART Predicts Immune Reconstitution on Therapy
Source: mBio. 2021 Mar 9;12(2):e00099-21. doi: 10.1128/mBio.00099-21 (PMC8092199; doi:10.1128/mBio.00099-21)
Supplement: TABLE S2 [file mBio.00099-21-st002.pdf]

Table S2. Biomarkers measured at 48 and 96 weeks of ART associated with the relative CD4+ counts at the corresponding time points.

**Biomarker**	**48 weeks ART**		**96 weeks ART**	
	rho	P[a]	rho	P
CD4 count	0.69	**4.19E-05**	0.76	**2.64E-06**
CD4/CD8 ratio	0.18	0.36	0.19	0.33
**Virological biomarkers**				
US RNA	0.03	0.89	0.14	0.48
MS RNA	0.12	0.54	0.06	0.76
Total DNA	-0.29	0.15	-0.01	0.97
US RNA / total DNA	0.24	0.23	0.26	0.19
US RNA / MS RNA	-0.31	0.13	0.15	0.45
**CD4+ T-cell subsets**				
CD4+ Tn	0.34	0.090	0.10	0.62
CD4+ Ttd	0.14	0.49	0.30	0.13
CD4+ Tcm	-0.02	0.92	-0.06	0.75
CD4+ Ttm	-0.27	0.17	-0.27	0.17
CD4+ Tem	-0.13	0.52	0.01	0.97
CD4+/CD31+/CD45RA+	0.20	0.30	-0.03	0.86
CD4+ Naive T-cell CD31+ subset	-0.04	0.82	-0.28	0.14
CD4+/Ki67+	0.21	0.28	0.02	0.91
Treg	-0.38	**0.049**	-0.29	0.14
**CD8+ T-cell subsets**				
CD8+ Tn	0.28	0.14	0.18	0.35
CD8+ Te	-0.04	0.86	-0.10	0.61
CD8+ Tcm	-0.07	0.71	0.23	0.24
CD8+ Ttm	-0.11	0.58	-0.02	0.93
CD8+ Tem	-0.14	0.48	-0.22	0.27
CD8+/ Ki67+	0.18	0.36	0.13	0.49
**CD4+ activation and exhaustion**				
CD4+/CD38+	0.10	0.60	0.22	0.27
CD4+/CD57+	-0.06	0.77	0.17	0.38
CD4+/CTLA-4+	0.22	0.25	0.04	0.85
CD4+/HLA-DR+	-0.24	0.21	0.03	0.88
CD4+/PD-1+	-0.06	0.77	0.06	0.74
CD4+/HLA-DR+/CD38+	-0.29	0.14	-0.06	0.75
CD4+/CTLA-4+/PD-1+	-0.04	0.83	-0.04	0.86
CD4+/CD57+/PD-1+	-0.13	0.50	0.12	0.54
CD4+/CD57+/HLA-DR+	-0.23	0.24	0.04	0.84
**CD4+ activation and exhaustion**				
CD8+/CD38+	-0.16	0.41	0.22	0.25
CD8+/CD57+	-0.03	0.88	-0.13	0.52
CD8+/CTLA-4+	0.03	0.89	0.09	0.64
CD8+/HLA-DR+	-0.29	0.13	0.04	0.83
CD8+/PD-1+	0.04	0.82	0.19	0.34

CD8+/HLA-DR+/CD38+	-0.25	0.21	0.14	0.46
CD8+/CTLA-4+/PD-1+	0.08	0.67	0.08	0.69
CD8+/CD57+/PD-1+	0.06	0.77	0.12	0.54
CD8+/CD57+/HLA-DR+	-0.16	0.42	-0.03	0.88
**CD4+ apoptosis**				
CD4+/ Annexin-V+	-0.04	0.85	0.28	0.14
CD4+FAS+	-0.14	0.49	-0.40	**0.037**
CD4+/AV+/FAS+	-0.15	0.45	-0.10	0.63
CD4+/AV+/CD38+	0.08	0.71	0.13	0.51
CD4+/AV+/HLA-DR+	-0.26	0.19	0.01	0.95
**CD8+ apoptosis**				
CD8+/ Annexin-V+	-0.22	0.26	0.41	**0.031**
CD8+FAS+	-0.03	0.90	0.11	0.59
CD8+/AV+/FAS+	-0.19	0.33	0.14	0.47
CD8+/AV+/CD38+	-0.15	0.45	0.39	**0.040**
CD8+/AV+/HLA-DR+	-0.33	0.088	0.09	0.67

[a] Significant P values are shown in bold type, those that remained significant after correction for multiple comparisons are underlined.
