# Supplementary material for: Cell-Associated HIV-1 Unspliced-to-Multiply-Spliced RNA Ratio at 12 Weeks of ART Predicts Immune Reconstitution on Therapy
Source: mBio. 2021 Mar 9;12(2):e00099-21. doi: 10.1128/mBio.00099-21 (PMC8092199; doi:10.1128/mBio.00099-21)
Supplement: TABLE S3 [file mBio.00099-21-st003.pdf]

Table S3. Predictive value of baseline biomarkers for the absolute and relative CD4+ count at 48 and 96 weeks of ART.

Biomarker	CD4+ count 48 weeks		Relative CD4+ count 48 weeks		CD4+ count 96 weeks		Relative CD4+ count 96 weeks	
	rho	P[a]	rho	P	rho	P	rho	P
CD4+ count	**0.64**	**2.23E-04**	0.02	0.92	**0.43**	**0.023**	-0.17	0.38
CD4/CD8 ratio	**0.43**	**0.024**	-0.11	0.60	0.38	0.051	-0.13	0.52
**Virological biomarkers**								
Plasma VL	0.23	0.23	**0.46**	**0.013**	0.16	0.42	**0.39**	**0.038**
US RNA	0.06	0.77	0.40	0.050	0.07	0.73	0.36	0.073
MS RNA	0.04	0.84	**0.41**	**0.034**	0.17	0.39	**0.46**	**0.015**
Total DNA	-0.03	0.90	-0.18	0.37	0.11	0.60	0.03	0.89
US RNA / total DNA	0.10	0.64	0.39	0.055	-0.03	0.88	0.19	0.35
US RNA / MS RNA	-0.13	0.53	-0.27	0.20	-0.28	0.17	-0.31	0.14
**CD4+ T-cell subsets**								
CD4+ Tn	0.28	0.23	0.27	0.26	-0.02	0.93	-0.01	0.96
CD4+ Ttd	0.20	0.39	0.31	0.19	0.26	0.27	0.36	0.12
CD4+ Tcm	0.13	0.52	-0.14	0.52	0.01	0.95	-0.23	0.26
CD4+ Ttm	-0.16	0.43	-0.09	0.68	-0.05	0.81	-0.07	0.75
CD4+ Tem	-0.34	0.095	-0.12	0.57	-0.03	0.90	0.17	0.41
CD4+/CD31+/CD45RA+	**0.55**	**0.0044**	0.22	0.29	0.02	0.93	-0.22	0.29
CD4+ naive T-cell CD31+ subset	0.39	0.056	0.33	0.12	0.00	0.99	0.00	0.99
CD4+/Ki67+	0.18	0.41	-0.15	0.48	0.19	0.36	-0.14	0.50
Treg	-0.15	0.44	-0.18	0.35	-0.12	0.53	-0.13	0.51
**CD8+ T-cell subsets**								
CD8+ Tn	0.35	0.088	0.09	0.67	0.02	0.91	-0.15	0.48
CD8+ Teff	-0.27	0.17	-0.06	0.77	-0.23	0.24	0.08	0.69
CD8+ Tcm	0.01	0.98	0.09	0.65	-0.15	0.45	-0.14	0.49
CD8+ Ttm	0.24	0.23	0.01	0.96	0.23	0.26	-0.09	0.67
CD8+ Tem	0.17	0.42	-0.06	0.76	**0.41**	**0.038**	0.09	0.65
CD8+/ Ki67+	-0.07	0.73	0.21	0.30	-0.11	0.58	0.15	0.46
**CD4+ activation and exhaustion**								
CD4+/CD38+	-0.02	0.93	-0.01	0.96	-0.15	0.45	-0.12	0.57
CD4+/CD57+	-0.28	0.16	-0.07	0.74	-0.16	0.44	0.06	0.79
CD4+/CTLA-4+	**0.42**	**0.034**	**0.40**	**0.044**	0.22	0.27	0.14	0.48
CD4+/HLA-DR+	-0.33	0.11	-0.31	0.13	-0.20	0.33	-0.17	0.42
CD4+/PD-1+	-0.34	0.088	-0.20	0.34	-0.17	0.42	-0.06	0.76
CD4+/HLA-DR+/CD38+	-0.22	0.27	-0.17	0.40	-0.20	0.34	-0.14	0.49
CD4+/CTLA-4+/PD-1+	0.08	0.68	0.17	0.40	0.03	0.87	0.08	0.69
CD4+/CD57+/PD-1+	-0.28	0.17	-0.09	0.65	-0.11	0.60	0.06	0.76
CD4+/CD57+/HLA-DR+	-0.08	0.69	-0.03	0.87	-0.09	0.67	-0.01	0.97
**CD8+ activation and exhaustion**								
CD8+/CD38+	-0.07	0.72	0.19	0.33	-0.05	0.81	0.16	0.40
CD8+/CD57+	-0.20	0.31	-0.24	0.22	0.08	0.67	0.14	0.49
CD8+/CTLA-4+	**0.45**	**0.016**	**0.44**	**0.019**	0.14	0.49	0.09	0.64

CD8+/HLA-DR+	-0.02	0.93	-0.08	0.68	0.06	0.75	-0.07	0.73
CD8+/PD-1+	-0.17	0.40	-0.16	0.42	-0.16	0.41	-0.14	0.48
CD8+/HLA-DR+/CD38+	0.04	0.86	0.05	0.81	0.08	0.67	0.01	0.95
CD8+/CTLA-4+/PD-1+	0.27	0.16	0.07	0.71	0.14	0.49	-0.04	0.82
CD8+/CD57+/PD-1+	0.02	0.90	-0.31	0.10	0.19	0.33	-0.13	0.51
CD8+/CD57+/HLA-DR+	0.07	0.73	-0.15	0.45	0.23	0.23	0.00	0.98
**CD4+ apoptosis**								
CD4+/Annexin-V+	0.06	0.76	0.35	0.075	0.05	0.80	0.27	0.19
CD4+FAS+	0.04	0.86	-0.09	0.65	0.17	0.40	-0.12	0.57
CD4+/Annexin-V+/FAS+	0.17	0.42	0.20	0.34	0.11	0.60	-0.03	0.88
CD4+/Annexin-V+/CD38+	0.03	0.87	0.31	0.12	0.04	0.86	0.26	0.19
CD4+/Annexin-V+/HLA-DR+	0.00	0.98	0.33	0.10	-0.03	0.87	0.17	0.40
**CD8+ apoptosis**								
CD8+/Annexin-V+	-0.12	0.56	0.22	0.25	0.02	0.93	0.27	0.16
CD8+FAS+	0.30	0.13	0.03	0.88	0.26	0.18	-0.09	0.65
CD8+/Annexin-V+/FAS+	0.05	0.81	0.15	0.44	0.02	0.90	0.04	0.86
CD8+/Annexin-V+/CD38+	-0.01	0.96	0.34	0.074	0.20	0.31	**0.49**	**0.0088**
CD8+/Annexin-V+/HLA-DR+	-0.03	0.88	0.22	0.26	0.01	0.95	0.09	0.65

[a] Significant P values are shown in bold type, those that remained significant after correction for multiple comparisons are underlined.
