# Supplementary material for: Cell-Associated HIV-1 Unspliced-to-Multiply-Spliced RNA Ratio at 12 Weeks of ART Predicts Immune Reconstitution on Therapy
Source: mBio. 2021 Mar 9;12(2):e00099-21. doi: 10.1128/mBio.00099-21 (PMC8092199; doi:10.1128/mBio.00099-21)
Supplement: TABLE S4 [file mBio.00099-21-st004.pdf]

Table S4. Immunological biomarkers associated with US RNA / MS RNA ratio at 12 weeks of ART.

Biomarker	rho	P[a]
CD4	-0.36	0.078
CD4/CD8 ratio	**-0.47**	**0.019**
**CD4+ T-cell subsets**		
CD4+ Tn	-0.35	0.11
CD4+ Ttd	0.08	0.72
CD4+ Tcm	-0.37	0.067
CD4+ Ttm	0.14	0.50
CD4+ Tem	**0.41**	**0.040**
CD4+/CD31+/CD45RA+	-0.40	0.050
CD4+ Naive T-cell CD31+ subset	0.06	0.78
CD4+/Ki67+	-0.10	0.65
Treg	0.15	0.48
**CD8+ T-cell subsets**		
CD8+ Tn	**-0.45**	**0.025**
CD8+ Te	0.38	0.064
CD8+ Tcm	-0.22	0.30
CD8+ Ttm	-0.21	0.32
CD8+ Tem	0.19	0.36
CD8+/ Ki67+	-0.19	0.35
**CD4+ activation and exhaustion**		
CD4+/CD38+	0.09	0.66
CD4+/CD57+	**0.42**	**0.039**
CD4+/CTLA-4+	-0.02	0.93
CD4+/HLA-DR+	**0.52**	**0.0071**
CD4+/PD-1+	0.29	0.15
CD4+/HLA-DR+/CD38+	**0.63**	**7.82E-04**
CD4+/CTLA-4+/PD-1+	0.34	0.10
CD4+/CD57+/PD-1+	**0.45**	**0.027**
CD4+/CD57+/HLA-DR+	**0.46**	**0.025**
**CD8+ activation and exhaustion**		
CD8+/CD38+	0.24	0.24
CD8+/CD57+	0.30	0.15
CD8+/CTLA-4+	-0.23	0.27
CD8+/HLA-DR+	0.39	0.051
CD8+/PD-1+	-0.06	0.78
CD8+/HLA-DR+/CD38+	0.39	0.056
CD8+/CTLA-4+/PD-1+	-0.14	0.50
CD8+/CD57+/PD-1+	0.06	0.77
CD8+/CD57+/HLA-DR+	0.21	0.32
**CD4+ apoptosis**		
CD4+/ Annexin-V+	**0.45**	**0.026**
CD4+FAS+	**0.46**	**0.022**
CD4+/AV+/FAS+	**0.59**	**0.0023**
CD4+/AV+/CD38+	**0.43**	**0.038**

CD4+/AV+/HLA-DR+	**0.57**	**<u>0.0034</u>**
**CD8+ apoptosis**		
CD8+/ Annexin-V+	0.29	0.18
CD8+FAS+	0.38	0.070
CD8+/AV+/FAS+	0.35	0.089
CD8+/AV+/CD38+	0.40	0.050
CD8+/AV+/HLA-DR+	0.36	0.082

[a] Significant P values are shown in bold type, those that remained significant after correction for multiple comparisons are underlined.
