# Supplementary material for: Cell-Associated HIV-1 Unspliced-to-Multiply-Spliced RNA Ratio at 12 Weeks of ART Predicts Immune Reconstitution on Therapy
Source: mBio. 2021 Mar 9;12(2):e00099-21. doi: 10.1128/mBio.00099-21 (PMC8092199; doi:10.1128/mBio.00099-21)
Supplement: TABLE S5 [file mBio.00099-21-st005.pdf]

Table S5. Antibody panels to measure the expression of immunological biomarkers.

**Panel 1**	**Fluorochrome**	**Dilution**
CD3	AlexaFluor700	50x
CD4	FITC	100x
CD8	APC-H7	50x
CD45RA	PE-Cy7	200x
CCR7	PE-CF594	100x
CD27	PerCP-Cy5.5	150x
CD31	PE	100x
Ki67	APC	100x

**Panel 2**	**Fluorochrome**	**Dilution**
CD3	AlexaFluor700	50x
CD4	FITC	100x
CD8	APC-H7	50x
CD38	PE-CF594	200x
HLA-DR	PE-Cy7	100x
CD57	PE	600x
PD-1	PerCP-Cy5.5	200x
CTLA-4	APC	100x

**Panel 3**	**Fluorochrome**	**Dilution**
CD3	AlexaFluor700	50x
CD4	FITC	100x
CD25	PE	100x
FoxP3	PerCP-Cy5.5	100x

**Panel 4**	Fluorochrome	Dilution
CD3	AlexaFluor700	50x
CD4	FITC	100x
CD8	APC-H7	50x
FAS	PE	100x
HLA-DR	PE-Cy7	100x
CD38	PE-CF594	200x
Annexin-V	APC	100x
